# Molecular Modeling of Vasodilatory Activity: Unveiling Novel Candidates Through Density Functional Theory, QSAR, and Molecular Dynamics

**DOI:** 10.3390/ijms252312649

**Published:** 2024-11-25

**Authors:** Anthony Bernal, Edgar A. Márquez, Máryury Flores-Sumoza, Sebastián A. Cuesta, José Ramón Mora, José L. Paz, Adel Mendoza-Mendoza, Juan Rodríguez-Macías, Franklin Salazar, Daniel Insuasty, Yovani Marrero-Ponce, Guillermin Agüero-Chapin, Virginia Flores-Morales, Domingo César Carrascal-Hernández

**Affiliations:** 1 Grupo de Investigaciones en Química y Biología, Departamento de Química y Biología, Facultad de Ciencias Básicas, Universidad del Norte, Carrera 51B, Km 5, vía Puerto Colombia, Barranquilla 081007, Colombia; awbernal@uninorte.edu.co (A.B.); insuastyd@uninorte.edu.co (D.I.); domingoh@uninorte.edu.co (D.C.C.-H.); 2 Facultad de Ciencias Básicas y Biomédicas, Programa de Química y Farmacia, Universidad Simón Bolívar, Carrera 59 N 59–65, Barranquilla 080002, Colombia; maryury.flores@unisimon.edu.co; 3 Grupo de Química Computacional y Teórica (QCT-USFQ), Departamento de Ingeniería Química, Universidad San Francisco de Quito, Diego de Robles y Vía Interoceánica, Quito 170901, Pichincha, Ecuador; sebastian.cuesta@postgrad.manchester.ac.uk (S.A.C.); jrmora@usfq.edu.ec (J.R.M.); 4 Department of Chemistry, Manchester Institute of Biotechnology, The University of Manchester, 131 Princess Street, Manchester M1 7DN, UK; 5 Departamento Académico de Química Inorgánica, Facultad de Química e Ingeniería Química, Universidad Nacional Mayor de San Marcos, Lima 15081, Peru; jpazr@unms.edu.pe; 6 Programa de Ingeniería Industrial, Universidad del Atlántico, Barranquilla 081007, Colombia; 7 Facultad de Ciencias de la Salud, Exactas y Naturales, Universidad Libre, Seccional Barranquilla, Barranquilla 080003, Colombia; 8 Centro de Química “Dr. Gabriel Chuchani”, Laboratorio de Síntesis Orgánica y Productos Naturales, Instituto Venezolano de Investigaciones Científicas (IVIC), Caracas 1020, Venezuela; fjosalazarr@gmail.com; 9 Facultad de Ingeniería, Universidad Panamericana, Augusto Rodin No. 498, Insurgentes Mixcoac, Benito Juárez, Ciudad de México 03920, Mexico; ymarrero@usfq.edu.ec; 10 Grupo de Medicina Molecular y Traslacional (MeM&T), Co-legio de Ciencias de la Salud (COCSA), Escuela de Medicina, Edificio de Especialidades Médicas, Diego de Robles y vía Interoceánica, Universidad San Francisco de Quito (USFQ), Quito 170157, Pichincha, Ecuador; 11 CIIMAR—Centro Interdisciplinar de Investigação Marinha e Ambiental, Universidade do Porto, Terminal de Cruzeiros do Porto de Leixões, Av. General Norton de Matos, s/n, 4450-208 Porto, Portugal; gchapin@ciimar.up.pt; 12 Departamento de Biología, Faculdade de Ciências, Universidade do Porto, Rua do Campo Alegre, s/n, 4169-007 Porto, Portugal; 13 Laboratorio de Síntesis Asimétrica y Bioenergética (LSAyB), Ingeniería Química (UACQ), Universidad Autónoma de Zacatecas, Campus XXI Km 6 Carr. Zac-Gdl, Zacatecas 98160, Mexico; virginia.flores@uaz.edu.mx

**Keywords:** cardiovascular diseases, vasodilators, QSAR modelling, molecular dynamics simulations, drug repurposing

## Abstract

Cardiovascular diseases (CVD) pose a significant global health challenge, requiring innovative therapeutic strategies. Vasodilators, which are central to vasodilation and blood pressure reduction, play a crucial role in cardiovascular treatment. This study integrates quantitative structure– (QSAR) modeling and molecular dynamics (MD) simulations to predict the biological activity and interactions of vasodilatory compounds with the aim to repurpose drugs already known and estimateing their potential use as vasodilators. By exploring molecular descriptors, such as electronegativity, softness, and highest occupied molecular orbital (HOMO) energy, this study identifies key structural features influencing vasodilatory effects, as it seems molecules with the same mechanism of actions present similar frontier orbitals pattern. The QSAR model was built using fifty-four Food Drugs Administration-approved (FDA-approved) compounds used in cardiovascular treatment and their activities in rat thoracic aortic rings; several molecular descriptors, such as electronic, thermodynamics, and topographic were used. The best QSAR model was validated through robust training and test dataset split, demonstrating high predictive accuracy in drug design. The validated model was applied on the FDA dataset and molecules in the application domain with high predicted activity were retrieved and filtered. Thirty molecules with the best-predicted pKI50 were further analyzed employing molecular orbital frontiers and classified as angiotensin-I or β1-adrenergic inhibitors; then, the best scoring values obtained from molecular docking were used to perform a molecular dynamics simulation, providing insight into the dynamic interactions between vasodilatory compounds and their targets, elucidating the strength and stability of these interactions over time. According to the binding energies results, this study identifies novel vasodilatory candidates where Dasabuvir and Sertindole seem to have potent and selective activity, offering promising avenues for the development of next-generation cardiovascular therapies. Finally, this research bridges computational modelling with experimental validation, providing valuable insight for the design of optimized vasodilatory agents to address critical unmet needs in cardiovascular medicine.

## 1. Introduction

Cardiovascular disease (CVD) is responsible for the highest number of deaths, causing approximately 17.9 million fatalities annually, accounting for 31% of all global deaths [1,2]. Along with mortality, this illness also causes significant morbidity and disability, burdening individuals, families, and healthcare systems. The World Health Organization (WHO) estimates that approximately 523 million people worldwide live with this disease [2,3]. Moreover, this number is expected to rise in the coming years, particularly in low- and middle-income countries. Therefore, effective prevention and management strategies are essential to reduce the impact of CVD on public health [3].

For decades, vasodilators have been used to treat various cardiovascular conditions by relaxing the smooth muscle cells in blood vessels, which causes an increase in their diameter and a reduction in blood pressure. Different categories of vasodilators include direct-acting vasodilators, calcium channel blockers, and nitric oxide (NO) donors, each with its mechanisms of action [4,5,6]. Despite their therapeutic benefits, vasodilators can cause adverse effects, such as headaches, flushing, and hypotension. Various factors, including molecular structure, pharmacokinetics, and target specificity, can influence the efficacy and safety of vasodilators. Therefore, the development of novel vasodilators with improved efficacy, selectivity, and safety profiles is necessary [7,8,9,10].

Structure–activity relationship (SAR) and quantitative structure–activity relationship (QSAR) studies have emerged as powerful tools for rationalizing and optimizing vasodilators. SAR involves the relationship between the structural features of a molecule and its biological activity. At the same time, QSAR uses computational methods to build predictive models of biological activity based on molecular descriptors and statistical algorithms [11,12,13,14]. Several SAR and QSAR studies have been conducted in recent years to investigate the vasodilatory activity of various compounds, including flavonoids, chalcones, quinazolines, pyrimidines and pyrazoles, guanidines, and pyrazolones. By analyzing the SAR and QSAR of these compounds, researchers have identified critical structural features responsible for their vasodilatory activity, such as specific functional groups, molecular size, and lipophilicity [15,16,17,18,19].

Recent advances in machine learning algorithms and high-throughput screening techniques have enabled the development of more accurate and predictive QSAR models. These models can be used to predict the biological activity of compounds based on their molecular descriptors, reducing the costs associated with traditional trial-and-error approaches and accelerating the drug discovery process [20,21].

In the context of vasodilators, QSAR models can identify novel compounds with potent and selective activity, using large datasets of experimental vasodilatory activity and molecular descriptors, including physicochemical properties, structural features, and pharmacokinetic parameters. The pool of FDA vasodilating compounds can be a potential source to build QSAR models, identify critical molecular determinants of vasodilatory activity, and develop more potent and selective compounds.

This study utilized density functional theory at ωB97XD/6−311++g (dp) level of theory to investigate several vasodilatory compounds already approved by the FDA. Electronic, topologic, and topographic molecular descriptors were generated to build a QSAR model. This model helped to identify potentially effective drugs with favorable binding energy profiles for repurposing. This work represents the first attempt to establish quantitative vasodilatory activity and drug-repurposing from FDA-approved compounds.

## 2. Results

### 2.1. Molecular Frontier Orbitals Analysis

Frontier orbitals play a fundamental role in understanding reaction mechanisms. Some articles have reported on the characteristics of these orbitals and their relationship with specific biological activities. For example, the relationship between the distribution of Highest occupied molecular orbital (HOMO) and The Lowest Unoccupied Molecular Orbita (LUMO) with antimicrobial activity has been described [22,23], as well as the nature of fluorescence in push–pull electron compounds [24,25,26]. For compounds with vasodilatory properties, this article represents the first attempt to find a relationship between this biological activity and the electronic distribution along the frontier orbitals.

**Figure 1:**
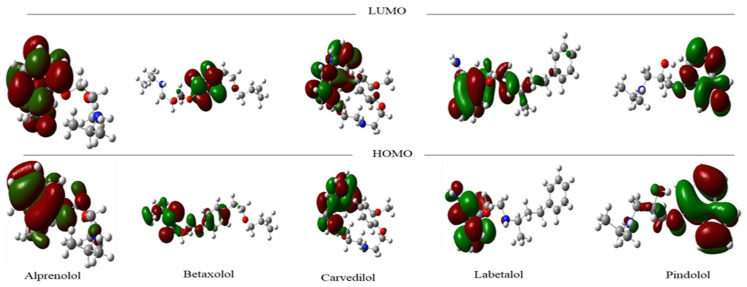
Frontier orbital for the five more active compounds against β1-adrenergic receptor.

Based on reported action mechanisms in the scientific literature, the data analyzed herein can be categorized into two distinct groups. The first group comprises β1-adrenergic receptor inhibitors, commonly referred to as beta-blockers [27]. These medications play a pivotal role in managing various conditions, including hypertension, angina pectoris, and cardiac arrhythmias. Beyond cardiovascular applications, beta-blockers also find utility in anxiety management, essential tremor mitigation, and ameliorating adverse effects associated with hyperthyroidism [28,29,30,31]. To explore potential correlation patterns between frontier orbitals and the inhibition of selected proteins, we selected the five most active compounds for each receptor from the available data (Table S1). Subsequently, we calculated the highest occupied molecular orbital (HOMO) and the lowest unoccupied molecular orbital (LUMO) (Figure 1). The frontier orbitals of Carvedilol, Pindolol, and Alprenolol (the most potent compounds) exhibit nearly overlapping highest occupied molecular orbital (HOMO) and lowest unoccupied molecular orbital (LUMO) regions within the molecular structure. By contrast, the less active compounds, Betaxolol and Labetalol, display discernible spatial separation between their HOMO and LUMO regions.

**Figure 2:**
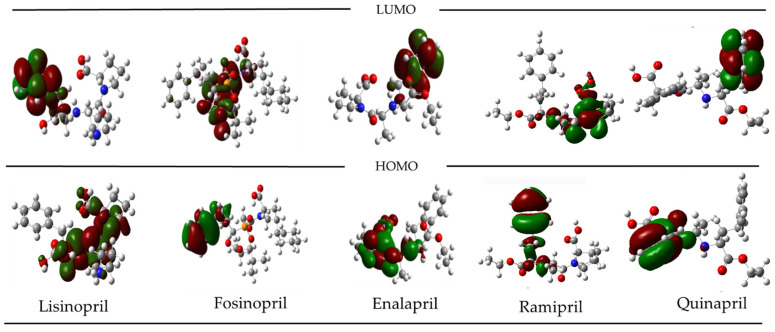
Frontier orbitals for the five more active compounds against angiotensin-converting enzyme.

The second group corresponds to angiotensin-converting enzyme (ACE) inhibitors, which contribute to blood pressure reduction by inducing relaxation in veins and arteries. Their mechanism involves inhibiting an enzyme responsible for producing angiotensin II within the body, a substance known to constrict blood vessels. By preventing this constriction, these inhibitors help to lower high blood pressure and reduce strain on the heart, which would otherwise need to work harder [32,33]. Figure 2 shows the five most active compounds against the angiotensin-converting enzyme (ACE). In contrast to the previous group (Figure 1), these compounds exhibit distinct characteristics in their HOMO and LUMO. Notably, both the HOMO and LUMO occupy opposite and well-defined regions within the molecule, implying specific electron-donating and electron-accepting regions. Although the qualitative nature of this finding is important, these unique characteristics provide a strong basis for preliminary screening. This can be followed by further evaluation of new compounds that may have vasodilatory effects.

### 2.2. QSAR Modelling

For the construction of the QSAR models, three types of descriptors were employed as follows: electronic descriptors, derived from conceptual density functional theory [34]; topological descriptors, derived from molecular graph theory [35,36]; and topographic descriptors. These latter molecular descriptors capture molecular information utilizing bilinear, quadratic, and linear algebraic forms, as well as theoretical edge-adjacency matrices and electronic density matrices. They consider both atom-based relations and bond-based relations within the molecule, thus qualifying as topographic descriptors [37].

Using these molecular descriptors as independent variables, and the value of KI50 as pKI50 (−log KI50) as the dependent variable, the following mathematical models were obtained.

1. pKI50 = 4.2275 + 2.1251A + 3.5830E − 05B − 10.1982C + 1.18754D − 1.0493E + 8.1419F + 0.6900G − 3.8395H; R^2^ = 0.8623, F = 33.088; s = 0.5846; N = 72; Q^2^loo = 0.7886; RMSE = 0.9377; MAE = 0.7941
2. pKI50 = 3.2838 +2.3428A + 0.0001B−11.0363C + 1.0286D−0.9335 E + 8.8309F + 0.8707G; R^2^ = 0.8307, F = 32.2440; s = 0.6241; N = 72; Q^2^loo = 0.7581; RMSE = 0.6741; MAE = 0.5431
3. pKI50 = 2.5933 + 2.3755A+ 0.0001B−0.3186C + 0.9599D + 8.3454E + 0.8515G R2 = 0.7876, F = 29.0428; s = 0.6916; N = 72; Q^2^loo = 0.7190; RMSE = 0.7421; MAE = 0.6035

where: A = VC_TrC_AB_nCi_3_M21 (M15)_SS1_T_LG3L [2−3]_LGL [2−3]_p_MID;

B = I50_TrF_AB_nCi_3_M20 (M10)_NS4_T_LG3P [1]_LGP [1]_h_MID;

C = MIC_SD_TrC_AB_nCi_3_M20 (M3)_SS0_X_KA_e_MID;

D = S_TrC_AB_nCi_3_M19 (M15)_SS7_T_LGTP [10−11]_h_MID

E = IB_VC_Tr_AB_nCi_3_M20 (M12)_NS7_T_LGBA [0.314−0.628]_psa-e-s_MID;

F = I50_TrF_AB_nCi_3_M21 (M11)_SS1_T_KA_h_MID;

G = MX_Tr_AB_nCi_3_M26 (M3)_NS7_X_LG3L [2−3]_LGL [2−3]_psa-e-v_MID

H = N2_TrC_AB_nCi_3_M26 (M15)_SS6_T_LGA [0.0−1.0]_h_MID

The topographic descriptors A-H include different types of metrics which are applied to the molecular graph, and the lower-case letters describe the physicochemical properties included in the calculation of the descriptor; that is: p for polarizability, h for hardness, e for electronegativity, s for softness, psa for polar surface area, and v for van der Waals volume. These physicochemical properties are atoms and bond weighted. Descriptions for each molecular descriptor can be found in the Supplementary Information (Table S1).

**Figure 3:**
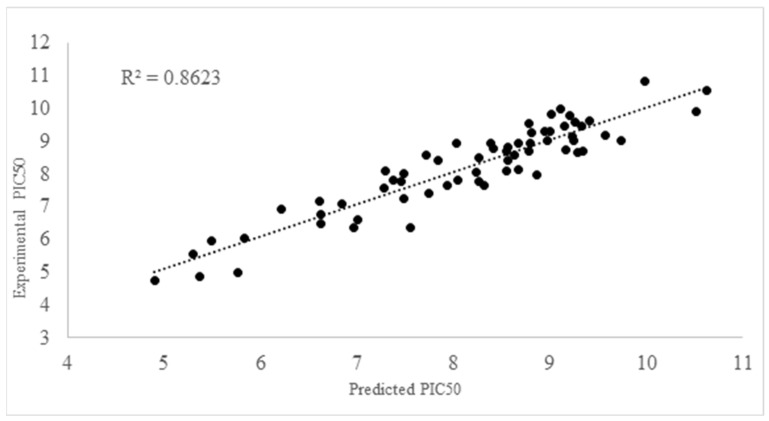
Experimental vs. predicted PKI50 values obtained from Model 1.

Model 1 stands out among the three models, capturing 85% of the observed variance and showing the best statistical performance. Table S2 shows the values of each descriptor in this model. It has the highest F-parameter, the lowest standard deviation, and excels in both internal and external validation. Figure 3 illustrates the strong correlation between experimental and predicted pKI50 values. Additionally, matrix correlations (Table S3) confirm no correlated molecular descriptors. Therefore, model 1 was selected for further analysis and evaluation.

According to Model 1, the descriptors A, D, F, and G have a positive influence on biological activity, while C and E, with negative coefficients, diminish this activity. A, D, F, and G involve several topological indices with electronic properties such as molecular polarizability; hardness, and polar surface areas, respectively; by contrast, C, E, and H have a negative influence on biological activity, with molecular descriptors such as electronegativity, softness, and HOMO energy.

To validate the external predictability of the model, the dataset was split into training (75%) and test sets (25%). Then, all the statistical parameters were re-calculated, and the results are presented in Table 1, by considering Tropsha’s test (available at www.oecd.org; accessed on 4 December 2023).

The evaluation of the applicability domain (AD) plays a crucial role in assessing the reliability of pKI50 predictions. It involves determining the theoretical spatial region defined by the descriptors utilized in the model. While there is no universally acknowledged best method for AD determination, several recognized approaches exist. In this study, as implemented in AMBIT discovery, we employed methods based on principal component analysis (PCA) range, Euclidean distance, city-block distance, and probability density. The PCA method analyzes the range of each descriptor and identifies molecules that fall outside this range as being outside the AD. For the Euclidean and city-block methods, distances from the centroid are calculated, and compounds are categorized as either within or outside the AD based on a specified cut-off value. The probability density method constructs a standard distribution using parametric techniques, enabling the identification of interior empty regions within the dataset distribution.

To achieve a definitive conclusion in this study, a consensus method was employed. A molecule is considered outside the applicability domain (AD) if it is classified as such by two or more AD assessment techniques. This consensus-based approach ensures a comprehensive assessment and enhances the reliability of the AD determination process. Then, as a result, it was found that for the case of the Range PCA method, molecules are “out” of the AD, but in the other three methods, these molecules are “in” the AD and, finally, by considering the consensus approach, all the molecules are into the applicability domain, implying a coverage of 100%.

### 2.3. Drug Bank 5.1.7 Screening

By analyzing the extensive collection of approved drugs in the FDA database, we aim to identify existing compounds with fewer side effects that can be repurposed to expand their therapeutic applications beyond their original indications. This methodology holds great promise for accelerating the drug development process, capitalizing on known safety profiles, and potentially reducing the costs associated with new drug development [38]. Our study explores the systematic analysis of FDA-approved drugs, their molecular targets, and relevant disease pathways to uncover potential matches and propose new therapeutic strategies. Through this approach, we aim to contribute to the advancement of drug repurposing as an efficient and effective strategy for addressing unmet medical needs and promote precision medicine.

Model 1 was employed to screen the drug bank database [39], which encompasses four distinct datasets, namely approved, experimental, nutraceutical, and withdrawn molecules. Upon applying the model, the output data underwent a curation process to eliminate organometallic compounds, inorganic salts, mixture substances, and the compound used as the training set. Subsequently, the AD of the model was assessed using the curated dataset, resulting in 449 compounds in it. The corresponding pKI50 values for these compounds can be found in the Supplementary Materials (Table S4).

Among the compounds within the AD, a total of 165 approved, 40 experimental, 7 nutraceuticals, and 32 withdrawn compounds were identified. The range of pKI50 values observed for these compounds ranged from 4.09 to 12.17. Notably, upon comparing this interval with the training data, it became evident that several compounds exhibited similar predicted activity to current drugs. Consequently, these compounds were selected for further analysis, considering their potential for enhanced biological activity.

Table 2 presents the optimal thirty compounds, accompanied by their predicted pKI50 values. Close inspection reveals that these 30 compounds span diverse pharmacological families, including anti-histaminic, anticancer, antibiotic, anti-inflammatory, antipsychotic, cardioprotective, antiviral, and anti-obesity agents. Notably, the model successfully identified structures with vasodilatory potential (shown in bold) even though these were absent from the training dataset due to the lack of KI50 values. This finding is of paramount significance, as it implies that the model demonstrates statistical robustness, rendering its application domain suitable for the discovery of novel compounds or the repurpose of previously reported ones with high potential for vasodilation activity.

Due to their known toxic effects, carcinogenic compounds were excluded from further analysis, and only compounds with antiallergic, psychoactive, and anti-inflammatory properties that had the potential to interact with any of the proteins mentioned in the previous section were selected.

### 2.4. Molecular Orbital Analysis for the Best Candidate from Drug Bank Repurpose

**Figure 4:**
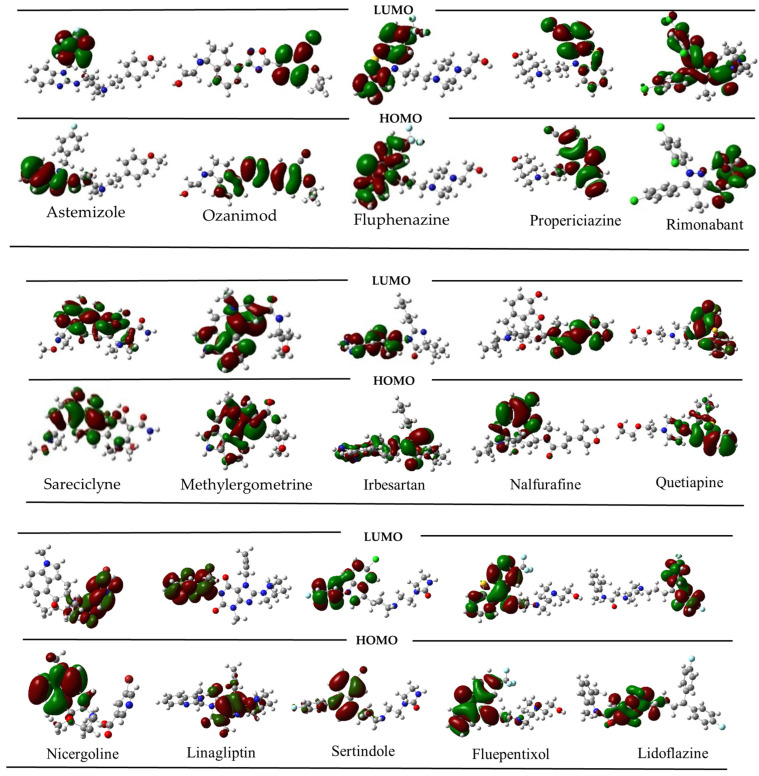
Frontier orbitals for selected drugs from the Drug bank using model 1. Up = LUMO; Down: HOMO.

Following the execution of a preliminary screening process encompassing a multitude of compounds, a subset of fifteen compounds with the most promising predicted activity were selected for subsequent optimization. This optimization was facilitated through the application of the ωB97XD/6−311++G (dp) theoretical level. The structures of these compounds corresponding to the minimum energy states were determined. Simultaneously, the frontier orbitals were analyzed, and the results were compared with the trends described in Section 2. This comparative study was designed to enhance our comprehension of the potential mechanistic pathways employed by each compound, as well as their prospective interactions with other molecular entities. Figure 4 offers a visual representation of the frontier orbitals associated with each compound, thereby elucidating their electronic characteristics and potential reactivity profiles.

Three distinct categories of compounds were discerned through the analysis. The first category encompasses Astemizole, Ozanimod, Fluphenazine, Propericiazine, Sarecycline, Methylergometrine, Irbesartan, Sertindole, Quetiapine, and Flupentixol. These compounds are characterized by the spatial arrangement of their HOMO (highest occupied molecular orbital) and LUMO (lowest unoccupied molecular orbital), which are situated on the identical side of the molecular structure. The second category, comprising Nalfurafine, Nicergoline, Linagliptin, and Lidoflazine, exhibits a contrasting orbital configuration, with the HOMO and LUMO located on disparate sides of the molecule. The third category is represented solely by Rimonabant, which does not conform to any discernible trend in terms of the spatial distribution of its HOMO and LUMO.

Interestingly, the first group of compounds, including Propericiazine, Quetiapine, Sertindole, Flupentixol, and Irbesartan, exhibit reported action against adrenergic receptors, which aligns with the suggestions made in Figure 1. Additionally, Astemizole and Fluphenazine have been reported as calcium and potassium channel inhibitors. On the other hand, Nalfurafine, Nicergoline, and Linagliptin, exhibit frontier orbitals localized in opposing regions of the molecular structure, as the behavior reported for ACE receptor inhibitors. Finally, Rimonabant, a compound with anti-inflammatory properties, does not display any of the characteristics described above, suggesting a possible distinct mechanism of action. These results indicate that the model can identify compounds exhibiting similar electronic behavior as those studied in the training data. Consequently, the analysis of frontier orbital shape is a promising starting point for selecting potential vasodilatory compounds.

### 2.5. Molecular Docking

#### Redocking

**Figure 5:**
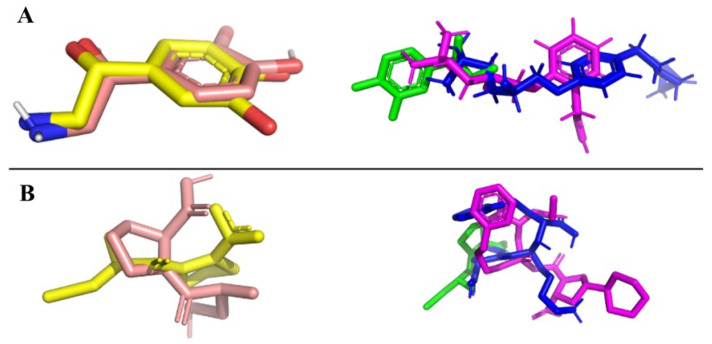
Redocking results. (**A**) Left: Noradrenaline redocking; Right: Noradrenaline (green), Alprenolol (blue), and Betaxolol (magenta); (**B**) Left: Captopril; Right: Captopril (green), Lisinopril (Azul), and Fosinopril (magenta).

Redocking is a computational technique used in molecular docking for drug design. Redocking allows researchers to estimate their confidence in the docking procedure they have conducted. Additionally, redocking is a validation technique [40,41]. The redocking images obtained for each protein are shown in Figure 5 (left). For each protein, a known inhibitor was used. The yellow structures correspond to the co-crystallized ligands (Norepinephrine, for adrenergic receptors, and Captopril, for ACE proteins, respectively) retrieved from the RSC PDB. The salmon-colored structures represent the poses obtained after the redocking protocol. The calculated root mean square deviation (RMSD) scoring values were 0.371 Å and 0.385 Å, respectively. These results suggest that these computational tools yield suitable approximations to experimental binding poses. Additionally, we docked each compound from a specific group against the corresponding named protein. Figure 5 (right) depicts the best-scoring compounds.

### 2.6. Molecular Docking for the Best Compounds Repurposing from the FDA Database

Following the validation of the protocol’s ability to generate docking poses akin to those observed in the protein data bank (PDB), we systematically docked thirty compounds derived from the FDA database repurposing against each target using consistent protocols. Notably, eight compounds failed to interact with the designated active sites of the selected targets. Consequently, Table 3 presents the scoring values exclusively for the remaining 22 compounds. According to Table 3, all 22 compounds exhibit scoring values equal to or greater than those obtained for the reference compounds. Furthermore, they display scores below −7, indicating a high non-covalent binding affinity for the inhibition of these [42]; moreover, in all cases, the RMSD was less than 2 Å, suggesting a suitable posing to coupling with the active site.

For the angiotensin-I receptor, Dasabuvir, Linagliptin, Idarubicin, and Rimonabant demonstrate scoring values twice that of the commercial compound Captopril. Finally, in the context of the β1-adrenergic receptor, four compounds demonstrate scoring values below −10: Dasabuvir, Lidoflazine, Niraparib, and Sertindole. Notably, Dasabuvir exhibits the most favorable performance across the two proteins studied herein, positioning this compound as a promising candidate for further experimentation in diverse assays.

**Figure 6:**
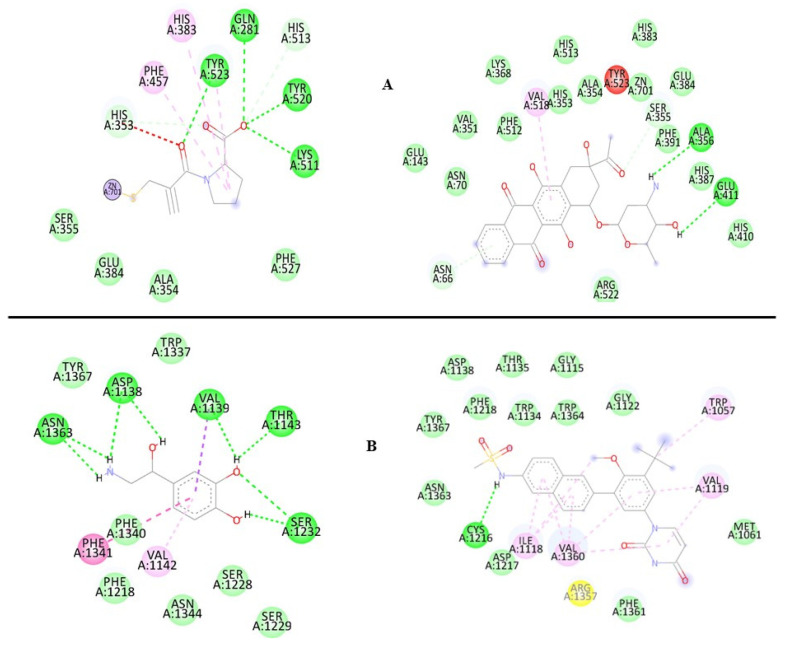
2D molecular diagram for the complex’s protein–ligand. (**A**) angiotensin-I receptor in complex with Captopril (left) and Idarubicin (right); (**B**) β1-adrenergic receptor in complex with Norepinephrine (left) and Dasabuvir (right).

To better understand how molecules interact and influence biological activity in our study, these 2D ligand–protein interaction diagrams were studied for each protein. We’ve highlighted the compound with the best scoring value in each case. For the angiotensin-I receptor (Figure 6A), we considered both Captopril and Idarubicin. We chose Idarubicin over Dasabuvir because it is already used in the context of the adrenergic receptor. Finally, for the adrenergic receptor (Figure 6B), we compared the complex with Norepinephrine and Dasabuvir.

Figure 6A depicts the 2D molecular interaction diagram between the angiotensin-converting enzyme (ACE) protein with Captopril (left) and the model-predicted compound, Idarubicin (right). Captopril’s mechanism of action is predominantly characterized by dipolar (VDW) interactions, with notable hydrogen bonds (HBs) involving residues Tyr523, Gln281, Tyr520, Lys511, and His353, all from chain A. Additionally, a cation–dipole interaction with the Zn^2+^ atom, deemed significant in the inhibition process [43]. Interestingly, idarubicin exhibits similar behavior, with most interactions being dipolar. Furthermore, direct interaction with the Zn^2+^ atom suggests a potential mechanism of action akin to the commercial compound Captopril. Conversely, the right side of the figure emphasizes the higher scoring value for Idarubicin compared to Captopril, attributed to an increased number of dipolar interactions with the active site, a consequence of its molecular structure.

Finally, Figure 6B presents the 2D diagrams of the β1-adrenergic receptor in complex with Norepinephrine (left) and Dasabuvir (right). Both compounds interact within the same binding pocket. However, Dasabuvir exhibits a greater number of dipolar molecular interactions, notably including a hydrogen bond with the Acys1216 residue absent in Norepinephrine. Additionally, Dasabuvir establishes several hydrophobic interactions with residues, such as Ilea1118, Val1360, Val1119, and Trp1057. The presence of aromatic rings, as well as carbonyl, sulfonic, and amine groups, facilitates molecular interactions, resulting in a lower scoring value.

### 2.7. Molecular Dynamics

**Figure 7:**
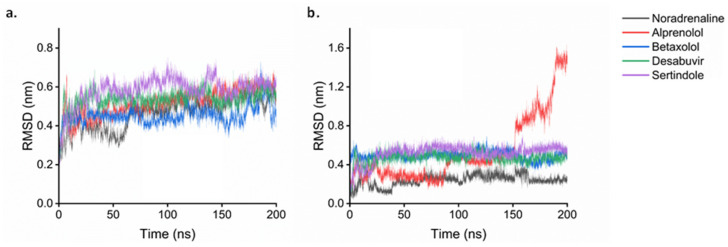
RMSD of the protein (**a**) and the ligand (**b**) in the MD simulation of β1-adrenergic receptor with various substrates.

**Figure 8:**
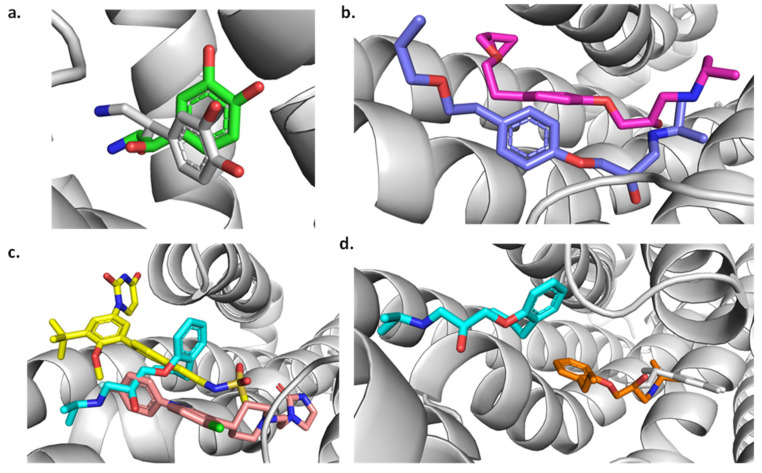
(**a**) Comparison of noradrenaline in the experimental conformation (white) vs. the result at the end of the MD simulation (green). (**b**) Comparison between the docking (blue) and the MD (pink) conformations of Betaxolol. (**c**) Dasabuvir (yellow), Sertindole (light pink), and Alprenolol (cyan) conformation after MD simulation. (**d**) docking (orange) and MD (cyan) conformation of Alprenolol in reference to Noradrenaline (white).

**Figure 9:**
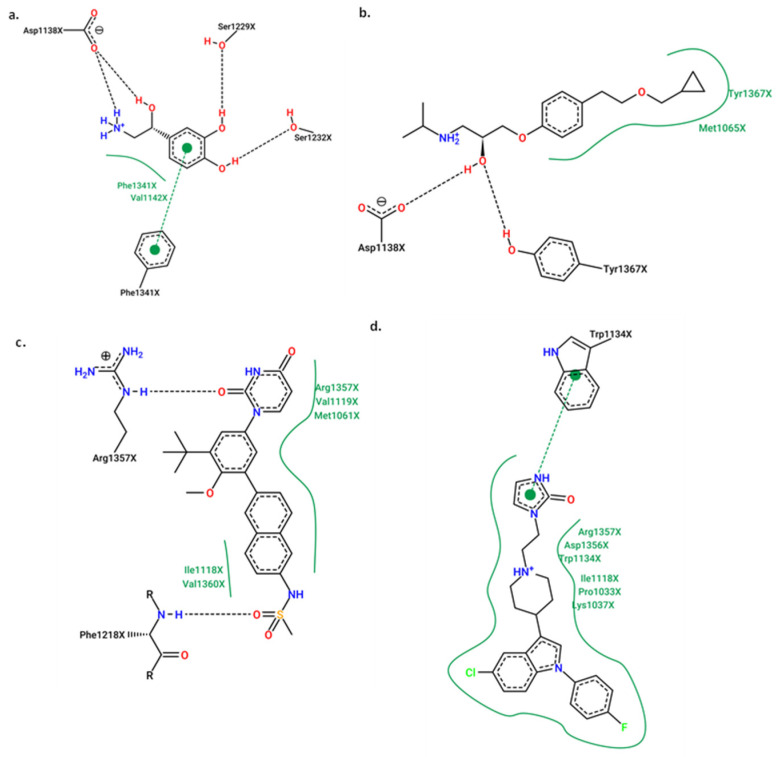
2D interactions of (**a**) Noradrenaline, (**b**) Betaxolol, (**c**) Dasabuvir, and (**d**) Sertindole.

The molecular dynamic simulations were divided into the two studied targets: β1-adrenergic receptor and angiotensin-I receptor. For each target, a 200 ns simulation was run as a control using the redocked conformation of the ligand that was co-crystallized experimentally. This was followed by the simulation of the other studied ligands. For the β1- adrenergic receptor, its complex with Alprenolol, Betaxolol, Dasabuvir, and Sertindole was simulated. The root mean square deviation (RMSD) analysis on the receptor shows the protein stays stable over time with values for all the systems of around 0.5 nm (Figure 7a). Noradrenaline and Betaxolol are the systems that present the lowest RMSD values in the receptor. Looking at the ligands RMSD (Figure 7b), all compounds present a value lower than 0.7 nm except Alprenolol which presents a maximum RMSD of 1.6 nm. An insight into the trajectory of the different systems (Figure 7) shows that in the case of Noradrenaline and Betaxolol, the ligands stayed inside the active site (Figure 8a,b). As expected, after the 200 ns, Noradrenaline almost overlaps its experimental conformation validating the calculations. For Betaxolol, its result suggests there is a possible rearrangement of the drug inside the active site without producing important changes in the receptor conformation. Dasabuvir and Sertindole (Figure 8c) appear in a similar conformation when comparing the start and the end of the dynamics, which is a result of strong interaction between the ligand and the protein. For Alprenolol (Figure 8d), the molecules moved to the entrance of the active site passing from being close of Betaxolol and Noradrenaline to be close to Dasabuvir and Sertindole (Figure 8d). Looking at the interactions (Figure 9), Noradrenaline and Betaxolol make an HB with Asp1138. Furthermore, Noradrenaline makes an HB with Ser1129 and Ser1232 while Betaxolol with Tyr1367. In the case of Dasabuvir and Sertindole, although their HBs are different between them, the hydrophobic interactions with Arg1357, Ile1118 are conserved. Free energy of binding (Table 4) shows all the values are negative, which translates to positive interactions between all these ligands and the receptor. Furthermore, the Betaxolol binding energy is comparable with the ones obtained for Dasabuvir and Sertindole, which suggests the inhibition of the receptor by these molecules may be feasible.

**Figure 10:**
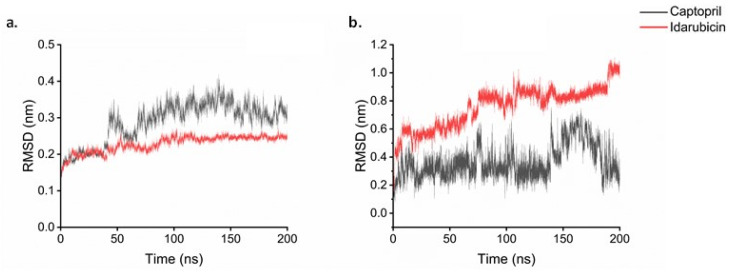
RMSD of the protein (**a**) and the ligand (**b**) in the MD simulation of β1-adrenergic receptor with various substrates.

**Figure 11:**
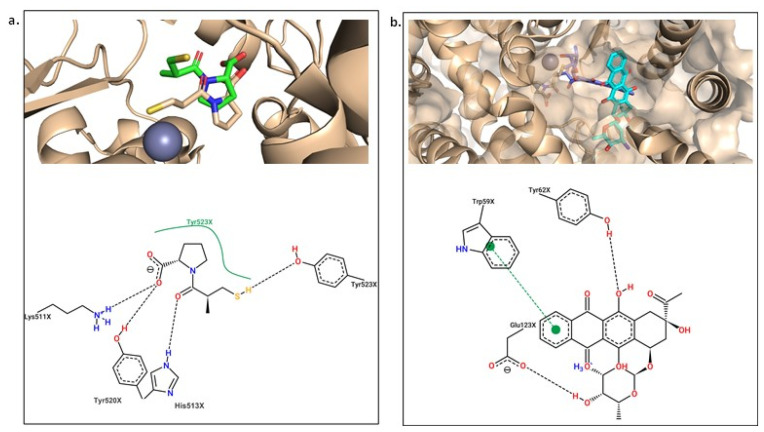
(**a**) Comparison of experimental (wheat) and MD (conformation) of Captopril. (**b**) Comparison of docked (blue) and MD (cyan) conformations of Idarubicin.

The simulations of the angiotensin-I receptor showed the protein is stable throughout the 200 ns with RMSD values of around 0.25 nm (Figure 10a). Analyzing the ligands (Figure 10b), Idarubicin RMSD value is around 0.4 nm higher than Captopril, which was expected, as Captopril is its known inhibitor. The same was found with the adrenergic receptor, the conformation of the co-crystalized ligand (Captopril) after the simulation agrees with the experimental crystal structure obtained (Figure 11a). For Idarubicin, it shows that the molecule has moved towards a chamber inside the active site where it keeps stable during the simulation (Figure 11b). Its interactions show HBs with Tyr62 and Glu123. Furthermore, there is a π − π stacking with Trp59. Its free energy of binding value (Table 4) is also negative suggesting that, although it is in a different part of the enzyme, Idarubicin is still making positive contacts with the receptor.

According to Table 4, Noradrenaline, a natural ligand of the adrenergic receptor, has a van der Waals energy of −22.91 and an electrostatic energy of −19.31. Betaxolol and Alprenolol, both commercial compounds with vasodilatory properties, have van der Waals energies of −47.54 and −29.69, respectively, and electrostatic energies of −12.72 and −10.62, respectively.

Dasabuvir, on the other hand, has a van der Waals energy of −53.62 and an electrostatic energy of −14.63. When comparing these values, we see that Dasabuvir has a more negative van der Waals energy than Noradrenaline, Betaxolol, and Alprenolol, suggesting a stronger interaction with the receptor. Its electrostatic energy is also more negative than Betaxolol and Alprenolol, but less negative than Noradrenaline.

On the other hand, Sertindole has a van der Waals energy of −54.71 and an electrostatic energy of −4.86. Idarubicin, on the other hand, has a van der Waals energy of −36.70 and an electrostatic energy of −9.32. When comparing these values, we see that Sertindole has a more negative van der Waals energy than Idarubicin, suggesting a stronger interaction with the receptor. Interestingly, Sertindole showed a binding energy similar to Captopril, a commercial vasodilator, suggesting future study of this compounds as a potential vasodilator.

In terms of binding energy, a more negative value indicates a stronger interaction between the compound and the receptor. This could suggest that Dasabuvir might have a strong affinity for the β1-adrenergic receptor while Sertindole and Idarubicin are for angiotensin I-receptor. So far, in line with the results obtained herein, the magnitude and sign of the binding energy suggest these three compounds seem to be the potential ones for treating cardiac disease via the vasodilatory mechanism. However, further research, including experimental studies, is needed to definitively determine whether these compounds have inhibitory effects on the adrenergic or angiotensin-I receptor, respectively.

## 3. Materials and Methods

### 3.1. Data Selection

Fifty-four compounds and their vasodilatory activities in rat thoracic aortic rings were taken from different databases, mainly from websites: PubChem [44], ChemSpider [45], Drug Bank [39] and the medchemexpress webpage (https://www.medchemexpress.com/ accessed on 17 August 2024). To maintain consistency in the data, the selected values must meet two criteria: first, the activity must be validated as an inhibitory constant of 50% (KI_50_) using the same protocol; and second, the compound must be commercially available or approved by the U.S. Food and Drug Administration (FDA). The smile structures for the whole data are depicted in the Supplementary Data (Table S5).

### 3.2. Molecular Descriptors Calculation

We obtained the structures of fifty-four selected compounds from the PubChem website (https://pubchem.ncbi.nlm.nih.gov/ accessed on 20 October 2024). First, these structures were processed using Avogadro software (1.99.0) [46] and a conformational analysis was carried out. Next, the structures were converted to gjf format, and their minimum energy was calculated using density functional theory, specifically the ωB97XD/6−311++G(dp) theory level. ωB97XD/6−311++G(d,p) was employed due to its extraordinary level of prediction shown in several QSAR models [47,48].

To ensure the accuracy of our results, we conducted frequency calculations on the energy-minimized structures of the fifty-four compounds. The structure was in an “energy minimum” if it had a complete set of real and positive values. These minimum-energy structures were then utilized to calculate a wide range of molecular descriptors, including the highest occupied molecular orbital (HOMO), lowest unoccupied molecular orbital (LUMO), polarizability (α), entropy (S), enthalpy (H), free energy (G), hardness (n), softness (s), electrophilic index (ω), lipophilicity (ClogP), polar surface area (psa), etc. Topological indexes, as well as all topographic descriptors were also obtained employing QuBiLS-MIDAS software 1.0 [49]. Molecular descriptors derived from conceptual DFT (µ, η, S, and ω) were calculated using Equations (1)–(4).

(1) μ=−x=−(IP+EA)/2

(2) η=(IP−EA)/2

(3) $$S=\frac{1}{2\eta }$$

(4) $$\omega =\frac{\mu^{2}}{2n}$$

### 3.3. QSAR Modeling Building

To enhance accuracy and alleviate overfitting caused by irrelevant or redundant [50] features, we adopted a descriptor selection approach. The Wrapper method, which can be applied to various machine-learning algorithms, identifies suitable descriptors for a subset [50]. This black-box technique scrutinizes the space to rank the features according to their predictive power, adding or removing them from the given subset. Using the software Weka 3.8 [51], we used multiple linear regression (MLR), partitioning the dataset into training (75%) and test (25%). The generated models were validated by applying different statistical techniques such as leave-one-out cross-validation, boot-trapping, and Y-scrambling, and the external validation was carried out by the mean of Tropsha’s test (available at www.oecd.org; accessed on 4 December 2023).

### 3.4. Applicability Domain

The applicability domain refers to the theoretical space within the training set of a model where QSAR predictions are deemed reliable. This domain is established based on the descriptors of the model. Defining the applicability domain enables the identification of outliers in the test set, i.e., compounds outside the domain whose prediction cannot be considered trustworthy. To define the applicability domain, we employed descriptors from the three most robust models and adopted a consensus approach, utilizing four methods (range, Euclidean distance, city-block distance, and probability density) implemented in AMBIT discovery v 0.04 (https://ambit.sourceforge.net/ accessed on 10 September 2024). If any outliers were detected, they were excluded from this study, and the statistical parameters of all models were recalculated accordingly [52,53].

### 3.5. Model Performance

The quality of the model was assessed based on both goodness-of-fit and goodness-of-prediction parameters. Goodness-of-fit parameters evaluate the degree of agreement between the sample data and the model and assess the model’s ability to account for the variance observed in the training set. We used two statistics to evaluate goodness-of-fit: the mean absolute error (MAE) and the coefficient of determination (R^2^). Goodness-of-prediction parameters, on the other hand, assess the model’s predictive power, both within the training set (using a tenfold cross-validation coefficient, Q^2^CV) and with external compounds that were not used to train the model (external validation coefficient, Q^2^_EXT_).

### 3.6. Molecular Docking

The molecular docking process was conducted to evaluate the binding interactions of selected compounds with the β1-adrenergic receptor and angiotensin-I-converting enzyme (ACE). The docking protocols were designed to ensure accurate representation of ligand poses in relation to the crystallized structures.

#### 3.6.1. Preparation of Protein Structures

The crystal structures of the β1-adrenergic receptor and ACE were retrieved from the Royal Society of chemistry protein data bank (www.rcsb.org, accessed 30 January 2024). All co-crystallized ligands, metals, sugars, solvents, and water molecules were removed to prepare the proteins for docking. For the β1-adrenergic receptor protein, the crystal structure with the PDB id: 7BU6 was utilized, with Norepinephrine as the bond [54]. Norepinephrine, an endogenous compound released by the adrenal medulla, serves as a crucial neurotransmitter in both the central and autonomic nervous systems. It acts as a precursor to Epinephrine and functions as the primary transmitter for most postganglionic sympathetic fibers. On the other hand, for the angiotensin-converting enzyme (ACE) the X-Ray diffraction structure with PDB ID: 1UZF was used; this structure has Captopril as a co-crystallized ligand. Captopril is classified as an ACE inhibitor and is commonly prescribed to manage essential or renovascular hypertension, congestive heart failure, left ventricular dysfunction that occurs after a heart attack, and nephropathy.

#### 3.6.2. Ligand Preparation

The ligands were obtained from the PubChem database and prepared using Avogadro software (1.99.0). The minimum energy conformers were optimized at the ωB97XD/6−311++G(dp) level of theory. These optimized structures were then used in the docking process.

#### 3.6.3. Redocking Procedure

Redocking protocols were initiated using crystallized complexes as reference compounds. These crystallized structures guided the docking process. Once the docking generated poses that closely matched the crystallized structures, the protocols were verified and validated. This validation enabled the protocols to be used for comparing other compounds that lack reported complexes with the specific protein. Known inhibitors, such as Norepinephrine for the β1-adrenergic receptor and Captopril for ACE, were employed as reference ligands. An RMSD cutoff of 2 Å was set for pose redundancy, enabling the identification and elimination of redundant poses.

#### 3.6.4. Docking Simulations

Docking simulations were conducted using AutoDock Vina, employing a grid box centered on the active site of the receptor with dimensions of 21 × 21 × 21 Å. The docking protocol allowed for full ligand flexibility and utilized default exhaustiveness settings to ensure comprehensive sampling of binding conformations. In every instance, the docking outcome was compared with the original structure from the pdb file, and only the pose with a root mean square deviation (RMSD) ≤ 2 was taken into account. It was confirmed that the protocol consistently produced an identical pose. Subsequently, additional potential compounds were subjected to docking against these proteins. The best scoring values in each protein were used for molecular dynamics simulations.

### 3.7. Molecular Dynamics

Molecular dynamics (MD) simulation was used to obtain insights in the interactions over time between compounds with vasodilatory activity and their possible targets. Furthermore, this technique was used to evaluate the strength of the interaction and how some of the compounds predicted to have vasodilatory activity may interact with possible targets. To have a guide for the activity, natural inhibitors were used as comparative as well as the best predicted P_KI50_ and the best scoring values in each target. For a starting conformation of the ligand in the target, the result of the docking calculations was considered. To build the topology of the two targets studied, the AMBER99SB-ILDN [55] force field was used. Moreover, for the ligands’ topology, the generalized amber force field (GAFF) [56] was chosen and obtained through the ACPYPE web server [57]. With the topology of the complex built, the system was solvated in a cube shape using a three-point water model (TIP3). Enough sodium or chlorine atoms were added to the solvent to neutralize the system. An energy minimization was performed followed by two equilibration steps of 100ps each. The first equilibration was performed at 300K using a constant number of particles, volume and temperature (NVT) [58]; while, in the second, the number of particles, pressure, and temperature (NPT) [59] was maintained. Finally, each system was run for 200 ns at 1 Barr, and 300 K. All calculations were performed using GROMACS 2019.6 software [60]. To ensure no errors were produced during the simulation, parameters such as box size, pressure, volume, density, and temperature were evaluated. Moreover, parameters such as the root-mean-square deviation, the interaction over time, and the stability of the compound inside the active site were evaluated. The free energy of binding was also computed by adding the potential energies obtained from bonded and non-bonded interactions (EMM), the polar solvation energy (Gpolar), and the non-polar solvation energy (Gnonpolar) using the molecular mechanics Poisson–Boltzmann surface area (MM-PBSA) method implemented in the g_mmpbsa tool [61].

## 4. Conclusions

This study underscores the significance of utilizing advanced computational techniques, such as quantitative structure–activity relationship (QSAR) modelling and molecular dynamics simulations, in the field of cardiovascular therapy, specifically in the design and evaluation of vasodilatory compounds. By employing these computational tools, the research successfully identified novel vasodilatory candidates with potent and selective activity, including Dasabuvir, Sertindole, Nicergoline, Flupentixol, Astemizole, Ozanimod, Fluphenazine, Propericiazine, Rimonabant, Methylergometrine, Irbesartan, Nalfurafine, Quetiapine, Linagliptin, and Sarecycline. These findings offer promising prospects for the development of next-generation cardiovascular therapies with improved efficacy and safety profiles. The validation of the QSAR model and the insights gained from molecular dynamics simulations highlight the potential of these methodologies in accelerating the drug discovery process and optimizing vasodilatory compounds. By emphasizing key structural features and dynamic molecular interactions, this study provides a valuable framework for rationalizing vasodilatory agents and addressing critical challenges in cardiovascular medicine. The identified compounds present opportunities for further preclinical and clinical investigations, paving the way for the advancement of cardiovascular therapy and the development of more effective treatment options for cardiovascular diseases.

## Figures and Tables

**Table 1:** Validation based on Tropsha’s test for model 1 with training-test partition.

Criterion	Cross-Validation		External Validation	
	Result	Assessment	Result	Assessment
R^2^ > 0.6	0.803	PASS	0.803	PASS
Q^2^Val > 0.5	0.730	PASS	0.724	PASS
(Q^2^Val − R0^2^)/Q^2^Val < 0.1	0.004	PASS	0.087	PASS
(Q^2^Val − R0′^2^)/Q^2^Val < 0.1	0.077	PASS	0.004	PASS
abs(R0^2^ − R0′^2^) < 0.1	0.054	PASS	0.060	PASS
0.85 < k < 1.15	0.997	PASS	0.996	PASS
0.85 < km < 1.15	0.994	PASS	0.999	PASS

**Table 2:** The most active compounds predicted by model 1.

Compound	Predicted P_KI50_	Compound	Predicted P_KI50_
Astemizole	12.17	Rimonabant	11.04
Ozanimod	11.36	Idarubicin	10.96
Fluphenazine	11.23	Topotecan	10.92
Propericiazine	11.12	Sarecycline	10.86
Lasofoxifene	11.11	Dasabuvir	10.81
Methylergometrine	10.51	Irbesartan	10.48
Ursodeoxycholic acid	10.47	Tedizolid	10.38
Oxandrolone	10.34	Deoxycholic acid	10.30
Gemifloxacin	10.30	Nalfurafine	10.23
Quetiapine	10.23	Nicergoline	10.19
Ethynyl estradiol	10.14	Niraparib	10.14
Megestrol	10.05	Domperidone	10.04
Linagliptin	9.98	Sertindole	9.97
Ribociclib	9.96	Flupentixol	9.96
Cholic acid	9.95	Lidoflazine	9.95

**Table 3:** Scoring values for the best 22 compounds predicted in this work against the three selected targets. Captopril, Verapamil, and Cyclazocine represent control molecules.

Molecule	Angiotensin-I- Converting	β-Adrenergic Receptor	Molecule	Angiotensin-I- Converting	β-Adrenergic Receptor
Captopril	−6.6	-	Megestrol	−9.2	−8.7
Cyclazocine	-	−8.5	Methylergometrine	−9.2	−9.1
Astemizole	−9.4	−9.9	Nalfurafine	−8.9	−8.9
Cholic Acid	−9.6	−8.9	Nicergoline	−9.6	−9.8
Dasabuvir	−10.6	−10.7	Niraparib	−9.2	−10.2
Flupentixol	−9.1	−9.7	Oxandrolone	−9.0	−9.5
Fluphenazine	−9.3	−9.1	Ozanimod	−9.4	−9.7
Irbesartan	−9.8	−9.6	Propericiazine	−9.3	−9.3
Lidoflazine	−9.8	−10.5	Ribociclib	−9.7	−9.4
Linagliptin	−10.3	−9.5	Rimonabant	−10.1	−9.5
Idarubicin	−10.5	−9.7	Sarecycline	−8.8	−9.3
			Sertindole	−9.7	−10.2

**Table 4:** Free energy of binding of the possible inhibitors of the β1-adrenergic receptor and angiotensin-I receptor, respectively.

Compound	van der Waals Energy	Electrostatic Energy	SASA Energy	Binding Energy
Noradrenaline	−22.91	−19.31	−2.66	−12.07
Betaxolol	−47.54	−12.72	−5.14	−29.96
Alprenolol	−29.69	−10.62	−4.00	−12.46
Dasabuvir	−53.62	−14.63	−5.76	−27.42
Sertindole	−54.71	−4.86	−5.53	−28.95
Captopril	−94.67	−59.49	−12.21	−25.14
Idarubicin	−36.70	−9.32	−4.38	−15.00

## Data Availability

Data is contained within the article and Supplementary Materials Figure S1 and Tables S1–S5.

## Supplementary Materials

The following supporting information can be downloaded at: https://www.mdpi.com/article/10.3390/ijms252312649/s1.
