# Supplementary material for: Molecular Modeling of Vasodilatory Activity: Unveiling Novel Candidates Through Density Functional Theory, QSAR, and Molecular Dynamics
Source: Int J Mol Sci. 2024 Nov 25;25(23):12649. doi: 10.3390/ijms252312649 (PMC11641664; doi:10.3390/ijms252312649)
Supplement: Supplementary file 1 [file ijms-25-12649-s001.zip › ijms-3281496-supplementary.pdf]

Figure S1: the thirteen most active compounds reporpousing fron FDA by mean of model 1.

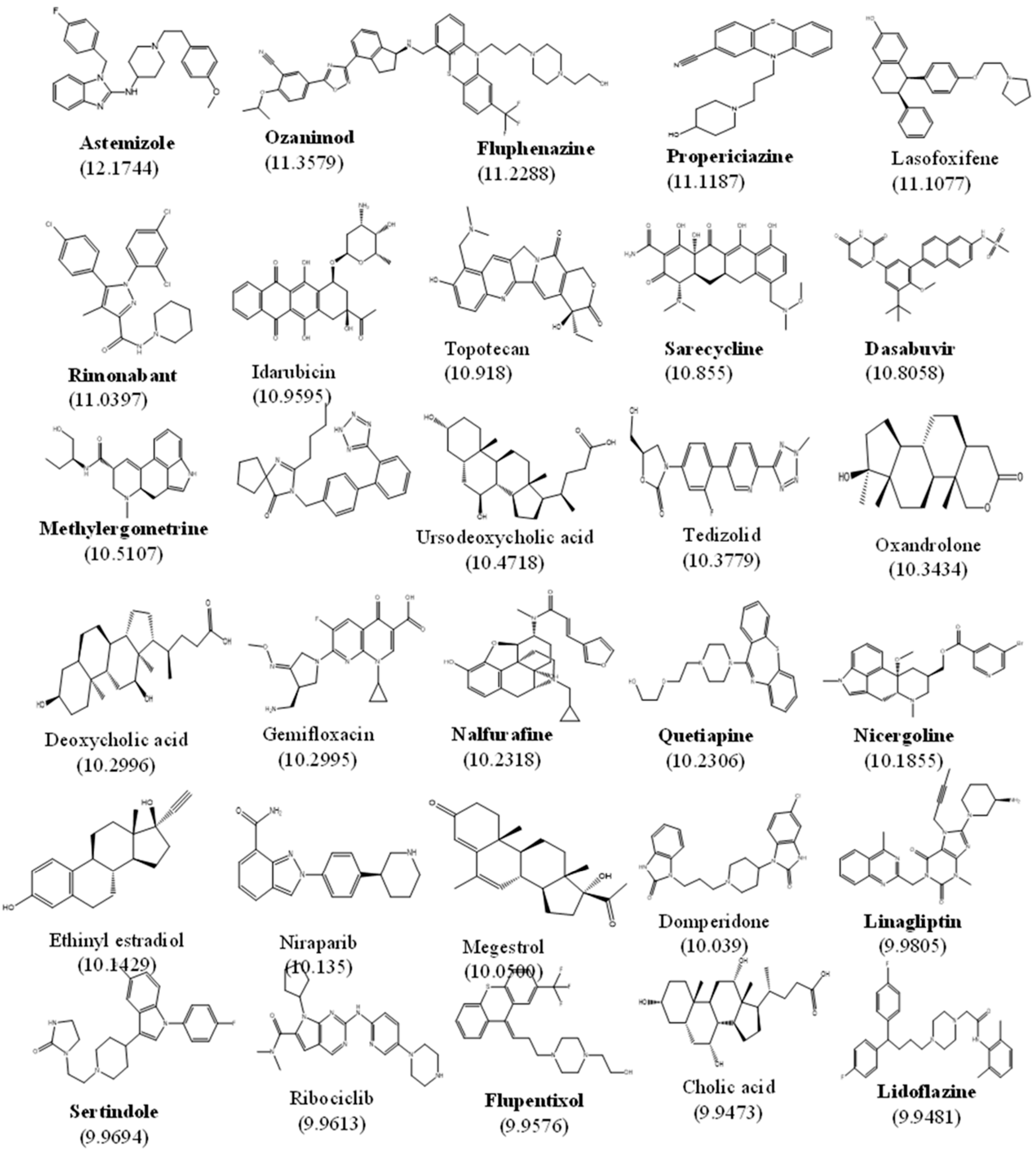

Table S1. Molecular Descriptor Definitions

Descriptor	Definition
A = VC_TrC_AB_nCi_3_M21(M15)_SS1_T_LG3L[2-3]_LGL[2-3]_p_MID	VC: Variation Coefficient Invariant<br>TrC: Three linear-cubic Indices

	AB: Atom-based Level<br>nCi: Non-chiral indices<br>3: Ternary<br>M21: Triangle area (Wave-Edges Distance)<br>SS: Simple Stochastic Matrix Order: 1<br>T: Total (Global) indices<br>LG3L: Ternary Cutoff based on Euclidean-Geometric Distance<br>Parameters: 2-3<br>LGL: Duplex Cutoff based on Euclidean-Geometric Distance<br>Parameters: 2-3<br>p: Polarisability
B = I50_TrF_AB_nCi_3_M20(M10)_NS4_T_LG3P[1]_LGP[1]_h_MID	I50: Q3-Q1 Invariant<br>TrF: Threelinear-linear Indices<br>AB: Atom-based Level<br>nCi: Non-chiral indices<br>3: Ternary<br>M20: Full Perimeter (Canberra Distance)<br>NS: Non Stochastic Matrix Order: 4<br>T: Total (Global) indices<br>LG3P: Ternary Cutoff based on Topological Distance<br>Parameters: 1<br>LGP: Duplex Cutoff based on Topological Distance<br>Parameters: 1<br>h: Hardness
C = MIC_SD_TrC_AB_nCi_3_M20(M3)_SS0_X_KA_e_MID	MIC: Mean Information Content Invariant<br>SD: Standard Deviation Invariant<br>TrC: Three linear-cubic Indices<br>AB: Atom-based Level<br>nCi: Non-chiral indices<br>3: Ternary

	M20: Full Perimeter (Minkowski Distance (Manhattan) p=1)<br>SS: Simple Stochastic Matrix Order: 0<br>X: Heteroatom (All atoms different to C and H atoms)<br>KA: Means keep all elements in the matrix form (entire matrix)<br>e: Electronegativity
D = S_TrC_AB_nCi_3_M19(M15)_SS7_T_LGTP[10-11]_h_MID	S: Skewness Invariant<br>TrC: Three linear-cubic Indices<br>AB: Atom-based Level<br>nCi: Non-chiral indices<br>3: Ternary<br>M19: Perimeter (Wave-Edges Distance)<br>SS: Simple Stochastic Matrix Order: 7<br>T: Total (Global) indices<br>LGTP: Ternary Cutoff based on Triangle Perimeter Multi-Metric<br>Parameters: 10-11<br>h: Hardness
E = IB_VC_Tr_AB_nCi_3_M20(M12)_NS7_T_LGBA[0.314-0.628]_psa-e-s_MID	IB: Ivanciuc - Balaban Invariant<br>VC: Variation Coefficient Invariant<br>Tr: Three linear Indices<br>AB: Atom-based Level<br>nCi: Non-chiral indices<br>3: Ternary<br>M20: Full Perimeter (Clark Distance)<br>NS: Non Stochastic Matrix Order: 7<br>T: Total (Global) indices<br>LGBA: Ternary Cutoff based on Bond Angle Multi-Metric<br>Parameters: 0.314-0.628<br>PSA: Topological Polar

	Surface Area<br>e: Electronegativity<br>s: Softness
F = I50_TrF_AB_nCi_3_M21(M11)_SS1_T_KA_h_MID	I50: Q3-Q1 Invariant<br>TrF: Three linear-linear Indices<br>AB: Atom-based Level<br>nCi: Non-chiral indices<br>3: Ternary<br>M21: Triangle area (Lance-Williams Distance)<br>SS: Simple Stochastic Matrix Order: 1<br>T: Total (Global) indices<br>KA: Means keep all elements in the matrix form (entire matrix)<br>h: Hardness
G = MX_Tr_AB_nCi_3_M26(M3)_NS7_X_LG3L[2-3]_LGL[2-3]_psa-e-v_MID	MX: Maximun Invariant<br>Tr: Threelinear Indices<br>AB: Atom-based Level<br>nCi: Non-chiral indices<br>3: Ternary<br>M26: Full Summation sides (Minkowski Distance (Manhattan) p=1)<br>NS: Non Stochastic Matrix Order: 7<br>X: Heteroatom (All atoms different to C and H atoms)<br>LG3L: Ternary Cutoff based on Euclidean-Geometric Distance<br>Parameters: 2-3<br>LGL: Duplex Cutoff based on Euclidean-Geometric Distance<br>Parameters: 2-3<br>psa: Topological Polar Surface Area<br>e: Electronegativity<br>v: Vdw Volume

H = N2_TrC_AB_nCi_3_M26(M15)_SS6_T_LGA[0.0-1.0]_h_MID	N2: Euclidean Distance Invariant<br>TrC: Three linear-cubic Indices<br>AB: Atom based Level<br>nCi: Non-chiral indices<br>3: Ternary<br>M26: Full Summation sides (Wave-Edges Distance)<br>SS: Simple Stochastic Matrix Order: 6<br>T: Total (Global) indices<br>LGA: Ternary Cutoff based on Triangle Area Multi-Metric<br>Parameters: 0.0-1.0<br>h: Hardness

Table S2. Molecular descriptors values used for model 1.

Molecule	pKIC50	A	B	C	d	e	F	G	H
Acebutolol	6.14	0.90	891316	1.27	0.09	4.56	2.85	0.17	8.09
Alprenolol	8.94	3.43	227899	1.31	0.09	10.71	4.68	0.04	10.81
Aspirin	8.49	7.78	41162	0.35	0.06	12.10	4.64	0.05	6.10
Betaxolol	7.43	1.49	475245	1.20	0.09	17.10	3.18	0.12	4.36
Captopril	9.92	6.72	57629	1.18	0.07	41.33	5.77	0.05	5.64
Carvedilol	9.75	3.68	1202642	2.13	0.09	23.07	2.50	0.82	6.10
Cilostazol	8.41	5.61	739687	0.54	0.13	15.89	3.26	0.12	12.28
Cinnarizine	6.79	0.14	796259	-0.08	0.12	21.27	4.28	0.11	0.00
Clevidipine	8.15	5.56	1453760	1.39	0.11	16.12	4.57	0.20	25.02
Clonidine	8.70	1.28	41807	-0.25	0.06	28.57	7.45	0.09	4.35
Diltiazem	7.55	5.58	922132	1.22	0.14	19.36	4.25	0.20	0.00
Dipyridamole	7.82	3.67	2027221	0.90	0.14	15.94	6.71	0.80	11.10
Doxazosin	9.13	6.57	1433183	0.88	0.12	24.38	3.04	0.87	24.23
Enalapril	8.92	8.16	989295	0.72	0.12	8.01	4.89	0.56	9.72
Eprosartan	8.70	6.25	1240809	0.71	0.11	22.37	6.01	0.23	16.89
Esmolol	6.71	2.20	527952	1.10	0.11	20.80	1.77	0.05	5.54
Felodipine	7.64	5.06	557641	1.04	0.12	17.12	3.61	0.08	8.02
Flunarizine	7.10	1.65	1091246	1.09	0.15	28.54	3.60	0.17	0.00
Fosinopril	9.00	6.98	4160245	2.28	0.17	15.29	3.31	0.58	24.39
Gallopamil 1	7.96	3.67	3524430	0.98	0.06	20.45	4.34	0.43	0.00
Hydralazine	6.05	4.23	18782	-0.13	0.11	6.25	3.48	0.04	0.92
Irbesartan	8.05	5.22	1188964	0.14	0.12	30.62	3.65	0.81	10.24
Isradipine	8.66	3.70	617583	1.15	0.09	25.83	4.74	0.11	16.15

Labetalol	8.00	4.39	636957	1.11	0.12	19.04	3.62	0.10	10.08
Levamlodipine	8.70	6.33	981585	1.32	0.12	28.65	4.35	0.12	7.82
Lidoflazine	7.80	2.77	2761175	1.57	0.06	29.40	4.14	0.14	4.89
Lisinopril	10.00	8.53	1355521	2.20	0.08	17.55	4.33	0.22	3.61
Metoprolol	6.75	2.97	322987	0.18	0.10	15.48	3.19	0.15	-0.33
Milrinone	6.91	7.37	76341	0.27	0.09	18.69	2.44	0.15	4.56
Moexipril	8.59	6.48	2860806	1.94	0.11	20.16	3.80	0.08	10.74
Nebivolol	6.50	1.86	990703	1.28	0.16	18.93	4.01	0.11	11.07
Nifedipine	9.00	9.26	549472	1.23	0.08	12.76	4.60	0.05	10.42
Nisoldipine	10.82	9.24	971135	0.38	0.13	12.84	6.90	0.95	19.22
Nitroglycerin	7.23	5.00	176061	1.04	0.11	3.10	3.50	0.07	13.54
Olmesartan	8.11	6.68	1565752	1.35	0.13	20.61	5.30	0.12	15.15
Papaverine	7.77	2.78	512615	1.82	0.13	23.45	2.45	0.13	0.00
Perhexiline	5.98	1.01	174744	0.06	0.10	14.24	2.24	0.20	10.48
Perindopril	8.82	8.51	771526	1.21	0.11	10.25	3.05	0.73	1.52
Phenoxybenzamine	5.57	2.06	319015	0.97	0.14	21.39	3.22	0.29	0.00
Pindolol	9.26	0.86	155129	1.10	0.09	29.35	4.61	0.28	13.59
Prazosin	9.77	5.29	754065	1.74	0.11	17.14	3.28	0.79	30.82
Prenylamine	7.19	1.24	565074	1.00	0.18	17.36	4.54	0.82	20.33
Quinapril	8.08	4.62	1650793	1.83	0.08	13.73	4.07	0.26	6.98
Ramipril	8.40	7.23	1210619	1.37	0.12	18.32	4.85	0.09	13.95
Sildenafil	9.30	6.87	1451240	1.51	0.13	17.05	6.40	0.22	11.96
Sotalol	6.36	4.16	226065	0.31	0.09	20.20	2.14	0.21	8.60
Telmisartan	9.48	3.10	2537868	0.93	0.07	25.05	5.82	0.21	34.17
Terazosin	7.77	5.88	754065	0.76	0.11	13.43	4.61	0.14	6.61
Tiapamil	4.87	5.04	3312490	1.21	0.19	18.88	3.19	0.22	0.00
Trandolapril	9.03	7.83	1404407	1.08	0.11	21.71	4.38	0.11	6.10
Valsartan	8.57	6.41	1682497	1.75	0.14	21.93	4.81	0.22	11.48
Vardenafil	10.54	8.79	1691134	1.63	0.09	21.62	3.72	0.68	7.11
Vinpocetine	7.41	1.24	346995	1.49	0.12	19.62	2.19	0.13	0.00
Zofenopril	9.40	7.46	1114723	0.52	0.13	25.26	4.34	0.14	10.59

Table S3. Correlation matrix between molecular descriptors in model 1.

	pKIC50	*A*	*B*	*C*	*D*	*E*	*F*	*G*
**pKIC50**	**1.00**							
**A**	**0.58**	**1.00**						
**B**	**0.14**	**0.20**	**1.00**					
**C**	**0.33**	**0.18**	**0.46**	**1.00**				
**D**	**-0.29**	**0.01**	**0.19**	**0.10**	**1.00**			
**E**	**0.24**	**-0.14**	**0.10**	**0.05**	**-0.11**	**1.00**		
**F**	**0.48**	**0.24**	**0.12**	**-0.13**	**-0.10**	**0.17**	**1.00**	
**G**	**0.35**	**0.19**	**0.25**	**0.09**	**0.22**	**-0.03**	**0.05**	**1.00**

Table S4. Predicted PIC50 for drug bank compounds and ISO-smiles codes.

pKIC50	SMILES	DRUG_GROUPS	GENERIC_NAME
12.1744	COC1=CC=C(CCN2CCC(CC2)NC2=NC3=CC=CC=C3N2CC2=CC=C(F)C=C2)C=C1	approved; withdrawn	Astemizole
11.3579	CC(C)OC1=C(C=C(C=C1)C1=NC(=NO1)C1=C2CC[C@H](NCCO)C2=CC=C1)C#N	approved; investigational	Ozanimod
11.2288	OCCN1CCN(CCCN2C3=CC=CC=C3SC3=C2C=C(C=C3)C(F)(F)F)CC1	approved	Fluphenazine
11.1187	OC1CCN(CCCN2C3=CC=CC=C3SC3=C2C=C(C=C3)C#N)CC1	approved; investigational	Periciazine
11.1077	[H][C@@]1(CCC2=CC(O)=CC=C2[C@@]1([H])C1=CC=C(OCCN2CCCC2)C=C1)C1=CC=CC=C1	approved; investigational	Lasofoxifene
11.0397	CC1=C(N(N=C1C(=O)NN1CCCCC1)C1=C(Cl)C=C(Cl)C=C1)C1=CC=C(Cl)C=C1	approved; investigational	Rimonabant
10.9595	C[C@@H]1O[C@H](C[C@H](N)[C@@H]1O)O[C@H]1C[C@@](O)(CC2=C1C(O)=C1C(=O)C3=CC=CC=C3C(=O)C1=C2O)C(C)=O	approved	Idarubicin
10.918	CC[C@@]1(O)C(=O)OCC2=C1C=C1N(CC3=CC4=C(C=CC(O)=C4CN(C)C)N=C13)C2=O	approved; investigational	Topotecan
10.855	[H][C@@]12CC3=C(CN(C)OC)C=CC(O)=C3C(=O)C1=C(O)[C@]1(O)C(=O)C(C(N)=O)=C(O)[C@@H](N(C)C)[C@]1([H])C2	approved; investigational	Sarecycline
10.8058	COC1=C(C=C(C=C1C1=CC2=CC=C(NS(C)(=O)=O)C=C2C=C1)N1C=CC(=O)NC1=O)C(C)(C)C	approved	Dasabuvir
10.5107	[H][C@@]12CC3=CNC4=CC=CC(=C34)C1=C[C@H](CN2C)C(=O)N[C@@H](CC)CO	approved	Methylergometrine
10.4746	CCCCC1=NC2(CCCC2)C(=O)N1CC1=CC=C(C=C1)C1=CC=CC=C1C1=NNN=N1	approved; investigational	Irbesartan
10.4718	[H][C@@]1(CC[C@@]2([H])[C@]3([H])[C@@H](O)C[C@]4([H])C[C@H](O)CC[C@]4(C)[C@@]3([H])CC[C@]12C)[C@H](C)CCC(O)=O	approved; investigational	Ursodeoxycholic acid
10.3779	CN1N=NC(=N1)C1=CC=C(C=N1)C1=CC=C(C=C1F)N1C[C@H](COP(O)(O)=O)OC1=O	approved	Tedizolid phosphate
10.3434	[H][C@@]12CC[C@](C)(O)[C@@]1(C)CC[C@@]1([H])[C@@]2([H])CC[C@@]2([H])CC(=O)OC[C@]12C	approved; investigational	Oxandrolone
10.2996	[H][C@@]12CC[C@H]([C@H](C)CCC(O)=O)[C@@]1(C)[C@@H](O)C[C@@]1([H])[C@@]2([H])CC[C@]2([H])C[C@H](O)CC[C@]12C	approved	Deoxycholic acid
10.2995	CO\N=C1/CN(CC1CN)C1=NC2=C(C=C1F)C(=O)C(=CN2C1CC1)C(O)=O	approved; investigational	Gemifloxacin

10.2318	CN([C@@H]1CC[C@@]2(O)[C@H]3CC4=CC=C(O)C5=C4[C@@]2(CCN3CC2CC2)[C@H]1O5)C(=O)\C=C\C1=COC=C1	experimental; investigational	Nalfurafine
10.2306	OCCOCCN1CCN(CC1)C1=NC2=CC=CC=C2SC2=CC=CC=C12	approved	Quetiapine
10.1855	[H][C@@]12CC3=CN(C)C4=CC=CC(=C34)[C@]1(C[C@@H](COC(=O)C1=CN=CC(Br)=C1)CN2C)OC	approved; investigational	Nicergoline
10.1429	[H][C@@]12CC[C@@](O)(C#C)[C@@]1(C)CC[C@]1([H])C3=C(CC[C@@]21[H])C=C(O)C=C3	approved	Ethinylestradiol
10.135	NC(=O)C1=CC=CC2=CN(N=C12)C1=CC=C(C=C1)[C@@H]1CCCNC1	approved; investigational	Niraparib
10.05	[H][C@@]12CC[C@](OC(C)=O)(C(C)=O)[C@@]1(C)CC[C@@]1([H])[C@@]2([H])C=C(C)C2=CC(=O)CC[C@]12C	approved; investigational; vet_approved	Megestrol acetate
10.039	ClC1=CC2=C(C=C1)N(C1CCN(CCCN3C(=O)NC4=CC=CC=C34)CC1)C(=O)N2	approved; investigational; vet_approved	Domperidone
9.9805	CC#CCN1C(=NC2=C1C(=O)N(CC1=NC3=C(C=CC=C3)C(C)=N1)C(=O)N2C)N1CCC[C@@H](N)C1	approved	Linagliptin
9.9694	FC1=CC=C(C=C1)N1C=C(C2CCN(CCN3CCNC3=O)CC2)C2=C1C=CC(Cl)=C2	approved; investigational; withdrawn	Sertindole
9.9613	CN(C)C(=O)C1=CC2=CN=C(NC3=CC=C(C=N3)N3CCNCC3)N=C2N1C1CCCC1	approved; investigational	Ribociclib
9.9576	[H]\C(CCN1CCN(CCO)CC1)=C1/C2=CC=CC=C2SC2=C1C=C(C=C2)C(F)(F)F	approved; investigational; withdrawn	Flupentixol
9.9473	[H][C@@](C)(CCC(O)=O)[C@@]1([H])CC[C@@]2([H])[C@]3([H])[C@]([H])(O)C[C@]4([H])C[C@]([H])(O)CC[C@]4(C)[C@@]3([H])C[C@]([H])(O)[C@]12C	approved	Cholic Acid
9.9421	CC1=CC=CC(C)=C1NC(=O)CN1CCN(CCCC(C2=CC=C(F)C=C2)C2=CC=C(F)C=C2)CC1	approved; experimental	Lidoflazine
9.9421	CC1=CC=CC(C)=C1NC(=O)CN1CCN(CCCC(C2=CC=C(F)C=C2)C2=CC=C(F)C=C2)CC1	approved; experimental	Lidoflazine
9.8784	[H][C@@]12C[C@@]3([H])[C@]4([H])C[C@H](F)C5=CC(=O)C=C[C@]5(C)[C@@]4(F)[C@@H](O)C[C@]3(C)[C@@]1(OC(C)(C)O2)C(=O)COC(C)=O	approved; investigational	Fluocinonide
9.8702	COC1=C(OCCCN2CCOCC2)C=C2C(NC3=CC(Cl)=C(F)C=C3)=NC=NC2=C1	approved; investigational	Gefitinib
9.8492	CN1CCN(CC1)C(=O)O[C@@H]1N(C(=O)C2=NC=CN=C12)C1=NC=C(Cl)C=C1	approved; investigational	Eszopiclone

9.8355	COC1=CC=C2N=C(COC3=CC=C(CC4SC(=O)NC4=O)C=C3)N(C)C2=C1	experimental; investigational	Rivoglitazone
9.8133	[H][C@@]12CC[C@@](O)(C#C)[C@@]1(CC)CC[C@]1([H])[C@@]3([H])CCC(C=C3CC[C@@]21[H])=NO	approved; investigational	Norelgestromin
9.7989	[H][C@@]12CC[C@H](OC(=O)CCCCCC)[C@@]1(C)CC[C@@]1([H])[C@@]2([H])CCC2=CC(=O)CC[C@]12C	approved	Testosterone enanthate
9.763	[H][C@@]12CC[C@@H](C)[C@]3([H])CC[C@@]4(C)OO[C@@]13[C@]([H])(O[C@@H](OC(=O)CCC(O)=O)[C@@H]2C)O4	approved; investigational	Artesunate
9.7258	C[C@]12CC[C@H]3[C@@H](CCC4=CC(=O)CC[C@]34C)[C@@H]1CC[C@@H]2OC(=O)CCC1CCCC1	approved	Testosterone cypionate
9.7199	NC1=NC(N2C=C(C(O)=O)C(=O)C3=CC(F)=C(N4CC(O)C4)C(Cl)=C23)=C(F)C=C1F	approved; investigational	Delafloxacin
9.6939	[H][C@]12CN3C4=C([C@@H](COC(N)=O)[C@@]3(OC)[C@@]1([H])N2)C(=O)C(N)=C(C)C4=O	approved	Mitomycin
9.6741	[H][C@@]12C[C@@H](C)[C@](O)(C(=O)CO)[C@@]1(C)C[C@H](O)[C@@]1([H])[C@@]2([H])[C@H](Cl)CC2=CC(=O)C=C[C@]12C	approved	Alclometasone
9.6691	[H][C@@]1(CC[C@@]2([H])[C@]3([H])C(=O)C[C@]4([H])CC(=O)CC[C@]4(C)[C@@]3([H])CC(=O)[C@]12C)[C@H](C)CCC(O)=O	approved; investigational	Dehydrocholic acid
9.6254	CCCCC1=NC(Cl)=C(CO)N1CC1=CC=C(C=C1)C1=CC=CC=C1C1=NNN=N1	approved	Losartan
9.6064	[H][C@@]12CC[C@](C)(O)[C@@]1(C)CC[C@@]1([H])[C@@]2([H])CCC2=CC(=O)CC[C@]12C	approved	Methyltestosterone
9.5876	[H][C@@]12C[C@@H](C)[C@](O)(C(=O)COC(C)=O)[C@@]1(C)C[C@H](O)[C@@]1([H])[C@@]2([H])C[C@H](F)C2=CC(=O)C=C[C@]12C	approved	Paramethasone acetate
9.585	[H][C@@]12CC[C@H]([C@H](C)CCC(=O)NCCS(O)(=O)=O)[C@@]1(C)CC[C@@]1([H])[C@@]2([H])[C@@H](O)C[C@]2([H])C[C@H](O)CC[C@]12C	experimental; investigational	Tauroursodeoxycholic acid
9.5807	[H][C@@]12CC[C@@](O)(C#C)[C@@]1(C)CC[C@]1([H])C3=C(CC[C@@]21[H])C=C(OC)C=C3	approved	Mestranol
9.5741	[H][C@@]12CC[C@](O)(C(=O)CO)[C@@]1(C)CC(=O)[C@@]1([H])[C@@]2([H])C[C@H](C)C2=CC(=O)C=C[C@]12C	approved; investigational	Methylprednisone
9.5712	COC1=CC=CC=C1OCCNCC(O)COC1=CC=CC2=C1C1=CC=CC=C1N2	approved; investigational	Carvedilol

9.5674	[H][C@@]12CC[C@](O)(C(=O)COP(O)(O)=O)[C@@]1(C)C[C@H](O)[C@@]1([H])[C@@]2([H])CCC2=CC(=O)C=C[C@]12C	approved; vet_approved	Prednisolone phosphate
9.565	[H][C@@]12CC[C@](O)(C(C)=O)[C@@]1(C)C[C@H](O)[C@@]1(F)[C@@]2([H])C[C@H](C)C2=CC(=O)C=C[C@]12C	approved; investigational	Fluorometholone
9.5619	CCOC(=O)N1CCC(CC1)=C1C2=C(CCC3=C1N=CC=C3)C=C(Cl)C=C2	approved; investigational	Loratadine
9.5532	[H][C@@]12OC3=C(O)C=CC4=C3[C@@]11CCN(CC3CC3)[C@]([H])(C4)[C@]1(O)CCC2=O	approved; investigational; vet_approved	Naltrexone
9.5393	CN(C1=CC2=NN(C)C(C)=C2C=C1)C1=CC=NC(NC2=CC=C(C)C(=C2)S(N)(=O)=O)=N1	approved	Pazopanib
9.5322	[H][C@]12O[C@@]1([H])[C@]1([H])C[C@@]([H])(C[C@@]2([H])[N+]1(C)C)OC(=O)C(O)(C1=CC=CS1)C1=CC=CS1	approved	Tiotropium
9.5244	COC1=C(OCC2CCN(C)CC2)C=C2N=CN=C(NC3=C(F)C=C(Br)C=C3)C2=C1	approved	Vandetanib
9.4873	CNCCCC12CCC(C3=CC=CC=C13)C1=CC=CC=C21	approved; investigational	Maprotiline
9.4803	[H][C@@]12CC[C@](C)(O)[C@@]1(C)CC[C@@]1([H])[C@@]2([H])CC[C@@]2([H])CC3=NNC=C3C[C@]12C	approved; vet_approved	Stanozolol
9.474	CC(C)(C)C1=CC=C(C=C1)C(=O)CCCN1CCC(CC1)OC(C1=CC=CC=C1)C1=CC=CC=C1	approved; investigational	Ebastine
9.4625	OC(=O)C1=CN(C2CC2)C2=CC(N3CCNCC3)=C(F)C=C2C1=O	approved; investigational	Ciprofloxacin
9.4473	[H][C@@]12CCC(=O)[C@@]1(C)CC[C@]1([H])C3=C(CC[C@@]21[H])C=C(OS(O)(=O)=O)C=C3	approved	Estrone sulfate
9.4229	CS(=O)(=O)C1=C2N(CC3=CC=C(Cl)C=C3)C3=C(CC[C@@H]3CC(O)=O)C2=CC(F)=C1	approved; investigational; withdrawn	Laropiprant
9.4125	[H][C@]12SCC(CSC3=NN=NN3C)=C(N1C(=O)[C@H]2NC(=O)[C@H](O)C1=CC=CC=C1)C(O)=O	approved; experimental	Cefamandole
9.4125	[H][C@]12SCC(CSC3=NN=NN3C)=C(N1C(=O)[C@H]2NC(=O)[C@H](O)C1=CC=CC=C1)C(O)=O	approved; experimental	Cefamandole
9.4112	COC1=C(OCCCN2CCOCC2)C=CC2=C1N=C(NC(=O)C1=CN=C(N)N=C1)N1CCN=C21	approved; investigational	Copanlisib
9.4059	COC1=C(OCCCN2CCC(CC2)C2=NOC3=C2C=CC(F)=C3)C=CC(=C1)C(C)=O	approved	Iloperidone

9.3965	[H][C@@]12CC[C@](O)(C(=O)COC(C)=O)[C@@]1(C)C[C@]([H])(O)[C@@]1([H])[C@@]2([H])CCC2=CC(=O)C=C[C@]12C	approved; vet_approved	Prednisolone acetate
9.3695	CC1=C(OC2=C(C=CC=C2C(=O)OCCN2CCCCC2)C1=O)C1=CC=CC=C1	approved	Flavoxate
9.3676	[H][C@@]1(OC(=O)C2=C1C=CC(OC)=C2OC)[C@]1([H])N(C)CCC2=CC3=C(OCO3)C(OC)=C12	approved; investigational	Noscapine
9.3641	[H][C@@]12CC[C@](OC(C)=O)(C(C)=O)[C@@]1(C)CC[C@@]1([H])[C@@]2([H])C[C@H](C)C2=CC(=O)CC[C@]12C	approved; investigational	Medroxyprogesterone acetate
9.3626	CN1CCN(CC1)C1=NC2=CC(Cl)=CC=C2NC2=CC=CC=C12	approved	Clozapine
9.3119	[H][C@@]1(CC[C@@]2([H])[C@]3([H])[C@H](O)C[C@]4([H])C[C@H](O)CC[C@]4(C)[C@@]3([H])C[C@H](O)[C@]12C)[C@H](C)CCC(=O)NCC([O-])=O	experimental; investigational	Glycocholic acid
9.3015	CC[C@@H]1CN(C[C@@H]1C1=CN=C2C=NC3=C(C=CN3)N12)C(=O)NCC(F)(F)F	approved; investigational	Upadacitinib
9.3013	OCCN1CCN(CC\C=C2\C3=C(SC4=C2C=C(Cl)C=C4)C=CC=C3)CC1	approved; investigational	Zuclopenthixol
9.2828	CCC1=NN(CCCN2CCN(CC2)C2=CC(Cl)=CC=C2)C(=O)N1CCOC1=CC=CC=C1	approved; withdrawn	Nefazodone
9.2693	[H][C@]1(C)C[C@@]2([H])[C@]3([H])CCC4=CC(=O)C=C[C@]4(C)[C@@]3([H])C(=O)C[C@]2(C)[C@@]1(O)C(=O)CO	approved; investigational	Meprednisone
9.2419	[H][C@@]12CC[C@@](O)(C#CC)[C@@]1(C)C[C@H](C1=CC=C(C=C1)N(C)C)C1=C3CCC(=O)C=C3CC[C@@]21[H]	approved; investigational	Mifepristone
9.2369	CC(C)[C@@H](C)\C=C\[C@@H](C)[C@H]1CC[C@H]2C3=CC=C4C[C@@H](O)CC[C@]4(C)[C@H]3CC[C@]12C	approved; experimental	Ergosterol
9.2369	CC(C)[C@@H](C)\C=C\[C@@H](C)[C@H]1CC[C@H]2C3=CC=C4C[C@@H](O)CC[C@]4(C)[C@H]3CC[C@]12C	approved; experimental	Ergosterol
9.2357	[H][C@@]12CC[C@@]3(CCC(=O)O3)[C@@]1(C)C[C@H]1O[C@@]11[C@@]2([H])[C@@H](CC2=CC(=O)CC[C@]12C)C(=O)OC	approved	Eplerenone
9.233	[H][C@@]12C[C@@H](C)[C@]34C=C(C)[C@H](OC(=O)C(\C)=C/C)[C@@]3(O)[C@H](O)C(CO)=C[C@@]([H])(C4=O)[C@]1([H])C2(C)C	approved	Ingenol mebutate
9.2301	[H][C@@]12CC[C@](O)(C(=O)CO)[C@@]1(C)C[C@H](O)[C@@]1([H])[C@@]2([H])C[C@H](F)C2=CC(=O)C=C[C@]12C	approved	Fluprednisolone

9.2269	C[C@H]1CN(C[C@@H](C)N1)C1=C(F)C(N)=C2C(=O)C(=CN(C3CC3)C2=C1F)C(O)=O	approved; investigational; withdrawn	Sparfloxacin
9.2133	[H][C@@]12C[C@]1([H])[C@@]1(C)C(=CC2=O)C(Cl)=C[C@@]2([H])[C@]3([H])CC[C@](OC(C)=O)(C(C)=O)[C@@]3(C)CC[C@]12[H]	approved; investigational	Cyproterone acetate
9.2007	[H][C@@]12CC[C@](O)(C(=O)COC(C)=O)[C@@]1(C)CC(=O)[C@@]1([H])[C@@]2([H])CCC2=CC(=O)C=C[C@]12C	approved; experimental; investigational	Prednisone acetate
9.2007	[H][C@@]12CC[C@](O)(C(=O)COC(C)=O)[C@@]1(C)CC(=O)[C@@]1([H])[C@@]2([H])CCC2=CC(=O)C=C[C@]12C	approved; experimental; investigational	Prednisone acetate
9.2004	[H][C@@]12CC[C@](O)(C(=O)CO)[C@@]1(C)CC(=O)[C@@]1([H])[C@@]2([H])CCC2=CC(=O)C=C[C@]12C	approved; vet_approved	Prednisone
9.199	CC1=C(C)C2=C(CCC(C)(COC3=CC=C(CC4SC(=O)NC4=O)C=C3)O2)C(C)=C1O	approved; investigational; withdrawn	Troglitazone
9.1989	[H][C@@]12CC[C@](O)(C(=O)COC(C)=O)[C@@]1(C)C[C@H](O)[C@@]1([H])[C@@]2([H])CCC2=CC(=O)CC[C@]12C	approved; vet_approved	Hydrocortisone acetate
9.1846	NC(=O)C1(CCN(CCC(C#N)(C2=CC=CC=C2)C2=CC=CC=C2)CC1)N1CCCCC1	approved; investigational	Piritramide
9.1692	CC(C)(C)C1=CC=C(CN2CCN(CC2)C(C2=CC=CC=C2)C2=CC=C(Cl)C=C2)C=C1	approved	Buclizine
9.168	[H][C@@]1(C)CCN(CCN1C(=O)C1=C(C=CC(C)=C1)N1N=CC=N1)C1=NC2=C(O1)C=CC(Cl)=C2	approved; investigational	Suvorexant
9.1642	[H][C@]1(C)C[C@@]2([H])[C@]3([H])CCC4=CC(=O)C=C[C@]4(C)[C@@]3(F)C(=O)C[C@]2(C)[C@@]1(O)C(=O)CCl	approved	Clobetasone
9.153	CCOC(=O)NC1=CC2=C(SC3=CC=CC=C3N2C(=O)CCN2CCOCC2)C=C1	approved; investigational; withdrawn	Moricizine
9.1405	[H][C@@]1(CC[C@@]2([H])[C@]3([H])CC[C@@]4([H])C[C@H](O)CC[C@]4(C)[C@@]3([H])CC[C@]12C)C(C)=O	approved; investigational	Brexanolone
9.1208	[H][C@@]12CC[C@@](O)(C#C)[C@@]1(C)CC[C@]1([H])C3=C(CC(=O)CC3)C[C@@]([H])(C)[C@@]21[H]	approved; investigational	Tibolone
9.1202	[H][C@@]1(CC[C@@]2([H])[C@]3([H])[C@H](O)C[C@]4([H])C[C@H](O)CC[C@]4(C)[C@@]3([H])CC[C@]12C)[C@H](C)CCC(O)=O	approved	Chenodeoxycholic acid

9.1175	OC1=C([C@H]2CC[C@@H](CC2)C2=CC=C(Cl)C=C2)C(=O)C2=CC=CC=C2C1=O	approved	Atovaquone
9.1172	O=C1CC2(CCCC2)CC(=O)N1CCCCN1CCN(CC1)C1=NC=CC=N1	approved; investigational	Buspirone
9.116	[H][C@@]12CC[C@](OC(=O)CCCC)(C(=O)CO)[C@@]1(C)C[C@H](O)[C@@]1([H])[C@@]2([H])CCC2=CC(=O)CC[C@]12C	approved; vet_approved	Hydrocortisone valerate
9.0947	CN(C)S(=O)(=O)C1=CC2=C(SC3=CC=CC=C3\C2=C\CCN2CCN(C)CC2)C=C1	approved	Thiothixene
9.0845	[H][C@@]12C[C@H](C)[C@](O)(C(=O)COP(O)(O)=O)[C@@]1(C)C[C@H](O)[C@@]1(F)[C@@]2([H])CCC2=CC(=O)C=C[C@]12C	approved; vet_approved	Betamethasone phosphate
9.0843	CCCC[N+]1(C)[C@H]2C[C@@H](C[C@@H]1[C@H]1O[C@@H]21)OC(=O)[C@H](CO)C1=CC=CC=C1	approved; investigational; vet_approved	Butylscopolamine
9.0785	CC#CC(=O)N1CCC[C@H]1C1=NC(=C2N1C=CN=C2N)C1=CC=C(C=C1)C(=O)NC1=CC=CC=N1	approved; investigational	Acalabrutinib
9.0695	C[C@@H]1C(=O)O[C@H]2C[C@]34[C@@H]5OC(=O)[C@]3(O[C@@H]3OC(=O)[C@H](O)[C@]43[C@@H]([C@H]5O)C(C)(C)C)[C@@]12O	nutraceutical	ginkgolide-J
9.0568	[H][C@@]12CC[C@](O)(C(=O)COC(=O)CC(C)(C)C)[C@@]1(C)C[C@H](O)[C@@]1([H])[C@@]2([H])CCC2=CC(=O)C=C[C@]12C	approved; vet_approved	Prednisolone tebutate
9.0556	[H][C@@]12CC[C@](O)(C(=O)COC(C)=O)[C@@]1(C)CC(=O)[C@@]1([H])[C@@]2([H])CCC2=CC(=O)CC[C@]12C	approved; investigational	Cortisone acetate
9.0549	[H][C@@]12C[C@@]3([H])[C@]4([H])C[C@H](F)C5=CC(=O)C=C[C@]5(C)[C@@]4(Cl)[C@@H](Cl)C[C@]3(C)[C@@]1(OC(C)(C)O2)C(=O)CO	approved; withdrawn	Fluclorolone acetonide
9.0524	[H][C@@]12CC[C@](O)(C(=O)COC(=O)CCC(O)=O)[C@@]1(C)C[C@H](O)[C@@]1([H])[C@@]2([H])CCC2=CC(=O)CC[C@]12C	approved	Hydrocortisone succinate
9.0406	[H][C@@]12CC[C@H](OC(=O)CC)[C@@]1(C)CC[C@@]1([H])[C@@]2([H])CCC2=CC(=O)CC[C@]12C	approved; investigational; vet_approved; withdrawn	Testosterone propionate
9.0239	CC1CN(CCN1)C1=C(F)C(C)=C2C(=O)C(=CN(C3CC3)C2=C1)C(O)=O	approved; investigational; withdrawn	Grepafloxacin
9.0203	CN1N=C(C(=O)N[C@@H]2C[C@@H]3CCC[C@H](C2)N3C)C2=C1C=CC=C2	approved; investigational	Granisetron

9.0142	[H][C@@]1(CC[C@@]2([H])[C@]3([H])[C@H](O)[C@H](CC)[C@]4([H])C[C@H](O)CC[C@]4(C)[C@@]3([H])CC[C@]12C)[C@H](C)CCC(O)=O	approved	Obeticholic acid
8.9977	C[C@H]1OC2=C(N)N=CC(=C2)C2=C(C#N)N(C)N=C2CN(C)C(=O)C2=C1C=C(F)C=C2	approved; investigational	Lorlatinib
8.98	CC1=NC(NC2=NC=C(S2)C(=O)NC2=C(C)C=CC=C2Cl)=CC(=N1)N1CCN(CCO)CC1	approved; investigational	Dasatinib
8.9778	[H][C@@]12CC[C@](OC(=O)OCC)(C(=O)COC(=O)CC)[C@@]1(C)C[C@H](O)[C@@]1([H])[C@@]2([H])CCC2=CC(=O)C=C[C@]12C	approved; investigational	Prednicarbate
8.9753	[H][C@@]12CC[C@](OC(=O)CCC)(C(=O)COC(=O)CC)[C@@]1(C)C[C@H](O)[C@@]1([H])[C@@]2([H])CCC2=CC(=O)CC[C@]12C	approved; vet_approved	Hydrocortisone probutate
8.97	COC1=CC(=CC(OC)=C1)N(CCNC(C)C)C1=CC=C2N=CC(=NC2=C1)C1=CN(C)N=C1	approved; investigational	Erdafitinib
8.9653	FC(F)(F)C1=CC=C(CNC2=NC=C(CC3=CNC4=NC=C(Cl)C=C34)C=C2)C=N1	approved; investigational	Pexidartinib
8.9502	CC1=CC(NC2=NC=NC3=CC=C(NC4=NC(C)(C)CO4)C=C23)=CC=C1OC1=CC2=NC=NN2C=C1	approved; investigational	Tucatinib
8.9467	FC1=CC=C(C=C1)C(=O)CCCN1CCC(=CC1)N1C(=O)NC2=CC=CC=C12	approved; vet_approved	Droperidol
8.9182	[H][C@@]12CC3=CN(C)C4=C3C(=CC=C4)C1=C[C@H](CN2C)C(=O)NC(CC)CO	approved	Methysergide
8.9167	CC1COC2=C3N1C=C(C(O)=O)C(=O)C3=CC(F)=C2N1CCN(C)CC1	approved	Ofloxacin
8.9155	O=C1CCC2=C(N1)C=CC(OCCCCC1=NN=NN1C1CCCCC1)=C2	approved; investigational	Cilostazol
8.9098	[H][C@@]12CCC(=O)[C@@]1(C)CC[C@@]1([H])[C@@]2([H])CC(=C)C2=CC(=O)C=C[C@]12C	approved; investigational	Exemestane
8.9064	[H][C@@]12CC[C@H](C(=O)NC(C)(C)C)[C@@]1(C)CC[C@@]1([H])[C@@]2([H])CC[C@@]2([H])NC(=O)C=C[C@]12C	approved	Finasteride
8.8787	FC1=CC=C(C=C1)C(CCCN1CCC(CC1)N1C(=O)NC2=CC=CC=C12)C1=CC=C(F)C=C1	approved	Pimozide
8.8767	CC1=CC=CC=C1C(=O)NC1=CC(C)=C(C=C1)C(=O)N1CCC[C@@H](O)C2=C1C=CC(Cl)=C2	approved	Tolvaptan
8.8699	[H][C@@]12C[C@@H](C)[C@](O)(C(=O)CO)[C@@]1(C)C[C@H](O)[C@@]1(F)[C@@]2([H])C[C@H](F)C2=CC(=O)C=C[C@]12C	approved; vet_approved	Flumethasone

8.8669	[H][C@@]12CC[C@](O)(C(=O)COC(=O)CCC(O)=O)[C@@]1(C)C[C@H](O)[C@@]1([H])[C@@]2([H])C[C@H](C)C2=CC(=O)C=C[C@]12C	approved	Methylprednisolone hemisuccinate
8.8668	[H][C@@]12CC[C@H](O)[C@@]1(C)CC[C@]1([H])[C@@]3([H])CCC(=O)C=C3CC[C@@]21[H]	experimental; investigational	Nandrolone
8.8615	CC1=NOC(NS(=O)(=O)C2=C(SC=C2)C(=O)CC2=CC3=C(OCO3)C=C2C)=C1Cl	approved; investigational; withdrawn	Sitaxentan
8.8449	CCCC1=NC(C)=C2N1NC(=NC2=O)C1=C(OCC)C=CC(=C1)S(=O)(=O)N1CCN(CC)CC1	approved	Vardenafil
8.8441	[H][C@@]12CC[C@H](O)[C@@]1(C)CC[C@]1([H])C3=C(CC[C@@]21[H])C=C(O)C=C3	approved; investigational; vet_approved	Estradiol
8.8257	CN1C=C(C=N1)C1=CN2N=CN=C(N3CCN(CC3)C3=NC=C(C=N3)[C@@](C)(N)C3=CC=C(F)C=C3)C2=C1	approved; investigational	Avapritinib
8.8217	[H][C@@]12C[C@@]3([H])[C@]4([H])CCC5=CC(=O)C=C[C@]5(C)[C@@]4([H])[C@@]([H])(O)C[C@]3(C)[C@@]1(N=C(C)O2)C(=O)COC(C)=O	approved; investigational	Deflazacort
8.7767	[H][C@@]12C[C@H](C)[C@](OC(=O)CC)(C(=O)CCl)[C@@]1(C)C[C@H](O)[C@@]1(F)[C@@]2([H])CCC2=CC(=O)C=C[C@]12C	approved	Clobetasol propionate
8.7655	[H][C@]12CN(C[C@]1([H])OCCN2)C1=C(F)C=C2C(=O)C(=CN(C3CC3)C2=C1C#N)C(O)=O	approved; investigational	Finafloxacin
8.7383	[H][C@@]12C[C@@H](C)[C@](O)(C(=O)CO)[C@@]1(C)C[C@H](O)[C@@]1(F)[C@@]2([H])CCC2=CC(=O)C=C[C@]12C	approved; investigational; vet_approved	Dexamethasone
8.722	[H][C@@]12CC[C@H](O)[C@@]1(C)CC[C@@]1([H])[C@@]2([H])CC[C@@]23O[C@@H]2C(O)=C(C[C@]13C)C#N	approved; investigational; vet_approved; withdrawn	Trilostane
8.7217	[H][C@@]12CC3=CNC4=CC=CC(=C34)C1=C[C@@H](CN2C)NC(=O)N(CC)CC	approved; investigational	Lisuride
8.7183	[H][C@@]12CCC3=CC(=O)C=C[C@]3(C)[C@@]1([H])CC[C@]1(C)OC(=O)CC[C@@]21[H]	approved; investigational	Testolactone
8.7174	CC(=O)[C@@]1(O)CC[C@H]2[C@@H]3C=C(C)C4=CC(=O)CC[C@@H]4[C@H]3CC[C@]12C	approved	Nomegestrol
8.7005	OC[C@H]1O[C@H]([C@H](O)[C@@H](O)[C@@H]1O)C1C2=CC=CC(O)=C2C(=O)C2=C1C=C(CO)C=C2O	approved; experimental	Alloin

8.7005	OC[C@H]1O[C@H]([C@H](O)[C@@H](O)[C@@H]1O)C1C2=CC=CC(O)=C2C(=O)C2=C1C=C(CO)C=C2O	approved; experimental	Alloin
8.6998	OS(=O)(=O)C1=CC2=C(N\C(C2=O)=C2\NC3=C(C=C(C=C3)S(O)(=O)=O)C2=O)C=C1	approved	Indigotindisulfonic acid
8.698	[H][C@@]12CC[C@H](C(C)=O)[C@@]1(C)CC[C@@]1([H])[C@@]2([H])CC=C2C[C@@H](O)CC[C@]12C	approved; experimental	Pregnenolone
8.698	[H][C@@]12CC[C@H](C(C)=O)[C@@]1(C)CC[C@@]1([H])[C@@]2([H])CC=C2C[C@@H](O)CC[C@]12C	approved; experimental	Pregnenolone
8.6964	CN(C)C1=CC2=C(C=C1)[N+](C)=C(\C=C\C1=C(C)N(C(C)=C1)C1=CC=CC=C1)C=C2	approved	Pyrvinium
8.6608	CN1CCN(CC1)C1=NC2=CC=CC=C2SC2=C1C=C(Cl)C=C2	approved	Clothiapine
8.6491	[H][C@@]12CC[C@@](O)(C#C)[C@@]1(C)CC[C@@]1([H])[C@@]2([H])CCC2=CC3=C(C[C@]12C)C=NO3	approved	Danazol
8.6483	CCSC1=CC2=C(SC3=CC=CC=C3N2CCCN2CCN(C)CC2)C=C1	approved; withdrawn	Thiethylperazine
8.6389	[H][C@@]12C[C@]3([H])C[C@@H](CC[C@@]3([H])[C@H](\C=C\C3=CC=C(C=N3)C3=CC(F)=CC=C3)[C@]1([H])[C@@H](C)OC2=O)NC(=O)OCC	approved	Vorapaxar
8.6387	[H][C@@]12C[C@H](C)[C@](O)(C(=O)CO)[C@@]1(C)C[C@H](O)[C@@]1(F)[C@@]2([H])C[C@H](F)C2=CC(=O)C=C[C@]12C	approved	Diflorasone
8.6305	CCC1=CC2=C(C=C1N1CCC(CC1)N1CCOCC1)C(C)(C)C1=C(C3=CC=C(C=C3N1)C#N)C2=O	approved; investigational	Alectinib
8.6269	COC1=C(NC(=O)\C=C\CN2CCCCC2)C=C2C(NC3=CC(Cl)=C(F)C=C3)=NC=NC2=C1	approved; investigational	Dacomitinib
8.623	CNC(=O)C1=CN(N=C1)C1=NC2=C(N=CN2[C@@H]2O[C@H](CO)[C@@H](O)[C@H]2O)C(N)=N1	approved; investigational	Regadenoson
8.6043	[H][C@@]12CC[C@](O)(C(=O)COC(=O)CCC3CCCC3)[C@@]1(C)C[C@H](O)[C@@]1([H])[C@@]2([H])CCC2=CC(=O)CC[C@]12C	approved; investigational; vet_approved	Hydrocortisone cypionate
8.6025	OC(=O)CSC1=NN=C(Br)N1C1=CC=C(C2CC2)C2=C1C=CC=C2	approved; investigational	Lesinurad
8.6011	C[C@@H]1CN(CC[C@]1(C(O)=O)C1=CC=CC=C1)[C@H]1CC[C@](CC1)(C#N)C1=CC=C(F)C=C1	approved; investigational	Levocabastine
8.6003	[H][C@@]12CC[C@@](O)(C#C)[C@@]1(CC)CC[C@]1([H])[C@@]3([H])CCC(=O)C=C3CC[C@@]21[H]	approved; investigational	Levonorgestrel

8.5965	[H][C@@]12[C@H]3CC[C@H](C3)[C@]1([H])C(=O)N(C[C@@H]1CCCC[C@H]1CN1CCN(CC1)C1=NSC3=CC=CC=C13)C2=O	approved; investigational	Lurasidone
8.5846	COC1=C2N(C=C(C(O)=O)C(=O)C2=CC(F)=C1N1CCNC(C)C1)C1CC1	approved; investigational	Gatifloxacin
8.5844	[H][C@@]12CC[C@H](OC(=O)CCCC)[C@@]1(C)CC[C@]1([H])C3=C(CC[C@@]21[H])C=C(O)C=C3	approved; investigational; vet_approved	Estradiol valerate
8.5734	[H][C@@]12CC[C@](O)(C(=O)COC(=O)CN(CC)CC)[C@@]1(C)C[C@H](O)[C@@]1([H])[C@@]2([H])CCC2=CC(=O)CC[C@]12C	approved	Hydrocortamate
8.5669	[H][C@@]12CC=C(C3=CC=CN=C3)[C@@]1(C)CC[C@@]1([H])[C@@]2([H])CC=C2C[C@@H](O)CC[C@]12C	approved	Abiraterone
8.5644	[H][C@]12CC[C@]([H])(C[C@@H](C1)OC(=O)C(O)(C1=CC=CC=C1)C1=CC=CC=C1)[N+]21CCCC1	approved	Trospium
8.5578	[H][C@@](CC)(NC1=NC=NC2=C1N=CN2)C1=NC2=CC=CC(F)=C2C(=O)N1C1=CC=CC=C1	approved	Idelalisib
8.5438	CC(=O)C1=C(C)C2=CN=C(NC3=NC=C(C=C3)N3CCNCC3)N=C2N(C2CCCC2)C1=O	approved; investigational	Palbociclib
8.5389	[H][C@](CO)(C(=O)O[C@@]1([H])C[C@@]2([H])[C@]3([H])O[C@]3([H])[C@@]([H])(C1)[N+]2(C)CC1CC1)C1=CC=CC=C1	experimental; investigational	Cimetropium
8.532	COC1=C(OC)C=C2C(N)=NC(=NC2=C1)N1CCN(CC1)C(=O)C1COC2=CC=CC=C2O1	approved	Doxazosin
8.5049	CN(C)C\C=C\C(=O)NC1=C(O[C@H]2CCOC2)C=C2N=CN=C(NC3=CC(Cl)=C(F)C=C3)C2=C1	approved	Afatinib
8.4759	CC(C)(C)C1=CC=C(C=C1)C(O)CCCN1CCC(CC1)C(O)(C1=CC=CC=C1)C1=CC=CC=C1	approved; withdrawn	Terfenadine
8.4733	O=C1N(C=C(C=C1C1=CC=CC=C1C#N)C1=NC=CC=C1)C1=CC=CC=C1	approved	Perampanel
8.469	[H][C@@]12C[C@@H](C)[C@](O)(C(=O)COC(C)=O)[C@@]1(C)C[C@H](O)[C@@]1(F)[C@@]2([H])CCC2=CC(=O)C=C[C@]12C	approved; investigational; vet_approved	Dexamethasone acetate
8.4656	[H][C@]12CN(C[C@@]1([H])NCCC2)C1=C(F)C=C2C(=O)C(=CN(C3CC3)C2=C1OC)C(O)=O	approved; investigational	Moxifloxacin
8.4653	C[N@+]1(CC2CC2)CC[C@]23[C@H]4OC5=C(O)C=CC(C[C@@H]1[C@]2(O)CCC4=O)=C35	approved	Methylnaltrexone
8.4625	[H][C@@]12CC[C@@](OC(C)=O)(C#C)[C@@]1(CC)CC[C@]1([H])[C@@]3([H])CC\C(C=C3CC[C@@]21[H])=N/O	approved; investigational	Norgestimate

8.4503	O=C1NC2=CC(OCCCCN3CCN(CC3)C3=C4C=CSC4=CC=C3)=CC=C2C=C1	approved; investigational	Brexpiprazole
8.4487	[H][C@@]12CC[C@@](O)(C#C)[C@@]1(C)CC[C@]1([H])C3=C(CC[C@@]21[H])CC(=O)CC3	approved	Norethynodrel
8.4458	[H][C@]1(O[C@H](CO)[C@@H](O)[C@H](O)[C@H]1O)C1=CC=C(C)C(CC2=CC=C(S2)C2=CC=C(F)C=C2)=C1	approved	Canagliflozin
8.4448	[H][C@@]12CC[C@@](O)(CCC(O)=O)[C@@]1(C)CC[C@@]1([H])[C@@]2([H])C=CC2=CC(=O)CC[C@]12C	approved; withdrawn	Canrenoic acid
8.4401	OC[C@H]1O[C@H]([C@H](O)[C@@H](O)[C@@H]1O)C1=CC=C(Cl)C(CC2=CC=C(O[C@H]3CCOC3)C=C2)=C1	approved	Empagliflozin
8.4181	[H][C@@]12C[C@@H](O)[C@H](O)[C@@]1(C)CC[C@]1([H])C3=C(CC[C@@]21[H])C=C(O)C=C3	approved; investigational; vet_approved	Estriol
8.4148	COC1=CC=C(C=C1)C1=C(OC2=CC=C(OCCN3CCCCC3)C=C2)C2=C(S1)C=C(O)C=C2	approved; investigational	Arzoxifene
8.3808	[H][C@@]12CC3=CNC4=CC=CC(=C34)C1=C[C@H](CN2C)C(=O)N[C@@H](C)CO	approved	Ergometrine
8.379	CN1N=CN=C1[C@@H]1[C@H](NC2=C3C1=NNC(=O)C3=CC(F)=C2)C1=CC=C(F)C=C1	approved; investigational	Talazoparib
8.3665	CN1CCN(CC1)C(=O)OC1N(C(=O)C2=NC=CN=C12)C1=NC=C(Cl)C=C1	approved	Zopiclone
8.3584	CN1CCN(CCCN2C3=CC=CC=C3SC3=C2C=C(Cl)C=C3)CC1	approved; vet_approved	Prochlorperazine
8.3549	[H][C@@]12CC[C@H](O)[C@@]1(C)CC[C@]1([H])C3=CC=C(OC(=O)N(CCCl)CCCl)C=C3CC[C@@]21[H]	approved; investigational	Estramustine
8.3471	[H][C@@]12CC[C@](O)(C(=O)CO)[C@@]1(C)C[C@H](O)[C@@]1([H])[C@@]2([H])CCC2=CC(=O)C=C[C@]12C	approved; vet_approved	Prednisolone
8.3465	[H][C@@]12C[C@@H](C)[C@](O)(C(=O)SCF)[C@@]1(C)C[C@H](O)[C@@]1(F)[C@@]2([H])C[C@H](F)C2=CC(=O)C=C[C@]12C	approved; experimental	Fluticasone
8.3465	[H][C@@]12C[C@@H](C)[C@](O)(C(=O)SCF)[C@@]1(C)C[C@H](O)[C@@]1(F)[C@@]2([H])C[C@H](F)C2=CC(=O)C=C[C@]12C	approved; experimental	Fluticasone
8.3374	FC(F)(F)C1=CC=C(C=C1)C(=O)NN1C(=O)[C@H]2[C@H]([C@H]3C=C[C@@H]2[C@@H]2C[C@H]32)C1=O	approved; investigational	Tecovirimat

8.3292	CSC1=CC2=C(SC3=CC=CC=C3N2CCC2CCCCN2C)C=C1	approved; withdrawn	Thioridazine
8.3105	CC1=NC2=C(CCN(C(=O)C3=CC=C(NC(=O)C4=CC=CC=C4C4=CC=CC=C4)C=C3)C3=CC=CC=C23)N1	approved; investigational	Conivaptan
8.2777	[H][C@@]12C[C@]1([H])N([C@@H](C2)C#N)C(=O)[C@@H](N)C12CC3CC(CC(O)(C3)C1)C2	approved	Saxagliptin
8.2758	CN1CCC(CC1)=C1C2=C(SC=C2)C(=O)CC2=CC=CC=C12	approved	Ketotifen
8.271	[H][C@](NC(=O)N1CCNC1=O)(C(=O)N[C@@H]1C(=O)N2[C@@H](C(O)=O)C(C)(C)S[C@]12[H])C1=CC=CC=C1	approved	Azlocillin
8.2616	[H][C@@]12[C@@H](O)[C@]3([H])C(=C)C4=C(C(O)=CC=C4)C(=O)C3=C(O)[C@]1(O)C(=O)C(C(N)=O)=C(O)[C@H]2N(C)C	approved; investigational	Metacycline
8.2604	[H][C@@]12CC[C@@](O)(C#C)[C@@]1(CC)CC(=C)[C@]1([H])[C@@]3([H])CCC(=O)C=C3CC[C@@]21[H]	approved; investigational	Etonogestrel
8.2576	N[C@@H]1CCCCN(C1)C1=C(F)C=C2C(=O)C(=CN(C3CC3)C2=C1Cl)C(O)=O	approved	Besifloxacin
8.252	NC1=NC2=C(N=CN2[C@@H]2C[C@H](CO)C=C2)C(NC2CC2)=N1	approved; investigational	Abacavir
8.2499	OC(C(=O)O[C@H]1C[N+]2(CCCOC3=CC=CC=C3)CCC1CC2)(C1=CC=CS1)C1=CC=CS1	approved	Aclidinium
8.2489	[H][C@@]12C[C@H]3OC(CCC)O[C@@]3(C(=O)CO)[C@@]1(C)C[C@H](O)[C@@]1([H])[C@@]2([H])CCC2=CC(=O)C=C[C@]12C	approved	Budesonide
8.246	NC1=NC=NC2=C1C(=NN2[C@@H]1CCCN(C1)C(=O)C=C)C1=CC=C(OC2=CC=CC=C2)C=C1	approved	Ibrutinib
8.2458	S(SC1=NC2=CC=CC=C2S1)C1=NC2=CC=CC=C2S1	approved; experimental	2,2'-Dibenzothiazyl disulfide
8.2458	S(SC1=NC2=CC=CC=C2S1)C1=NC2=CC=CC=C2S1	approved; experimental	2,2'-Dibenzothiazyl disulfide
8.2421	CN(C)S(=O)(=O)C1=CC2=C(SC3=CC=CC=C3N2CCCN2CCC(CCO)CC2)C=C1	approved; investigational	Pipotiazine
8.2335	[H][C@@]12CC[C@](OC(=O)CCC)(C(=O)CO)[C@@]1(C)C[C@H](O)[C@@]1([H])[C@@]2([H])CCC2=CC(=O)CC[C@]12C	approved; vet_approved	Hydrocortisone butyrate
8.2283	O=C1N(CC2=CC=CC=C2)C2C[S+]3CCCC3C2N1CC1=CC=CC=C1	approved; investigational	Trimethaphan

8.2188	[H][C@@]1(C)CC[C@@]2([H])[C@@]([H])(C)C([H])(O)O[C@]3([H])O[C@@]4(C)CC[C@]1([H])[C@@]23OO4	experimental; investigational	Artenimol
8.2055	[H][C@@]12C[C@H](C)[C@](O)(C(=O)CCl)[C@@]1(C)C[C@H](O)[C@@]1(F)[C@@]2([H])CCC2=CC(=O)C=C[C@]12C	approved; experimental; investigational	Clobetasol
8.2055	[H][C@@]12C[C@H](C)[C@](O)(C(=O)CCl)[C@@]1(C)C[C@H](O)[C@@]1(F)[C@@]2([H])CCC2=CC(=O)C=C[C@]12C	approved; experimental; investigational	Clobetasol
8.2047	ClC1=CC(Cl)=C(C=C1)C(CN1C=CN=C1)OCC1=CSC2=C1C=CC=C2Cl	approved; investigational	Sertaconazole
8.2045	CCCC1=NN(C)C2=C1N=C(NC2=O)C1=CC(=CC=C1OCC)S(=O)(=O)N1CCN(C)CC1	approved; investigational	Sildenafil
8.1947	FC(F)(F)C1=CC(=CC=C1)N1CCN(CCN2C(=O)NC3=CC=CC=C23)CC1	approved; investigational	Flibanserin
8.1855	[H][C@@]12CC[C@](O)(C(=O)COP(O)(O)=O)[C@@]1(C)C[C@H](O)[C@@]1([H])[C@@]2([H])CCC2=CC(=O)CC[C@]12C	approved; vet_approved	Hydrocortisone phosphate
8.1753	[H][C@@]12CC(=C)[C@](OC(C)=O)(C(C)=O)[C@@]1(C)CC[C@]1([H])[C@@]3([H])CCC(=O)C=C3CC[C@@]21[H]	approved; experimental; investigational	Segesterone acetate
8.1753	[H][C@@]12CC(=C)[C@](OC(C)=O)(C(C)=O)[C@@]1(C)CC[C@]1([H])[C@@]3([H])CCC(=O)C=C3CC[C@@]21[H]	approved; experimental; investigational	Segesterone acetate
8.1748	CC(=O)C1=CC=C2SC3=C(C=CC=C3)N(CCCN3CCN(CCO)CC3)C2=C1	approved	Acetophenazine
8.17	CN1CCN(CCCN2C3=CC=CC=C3SC3=CC=CC=C23)CC1	approved; investigational	Perazine
8.1632	OC(COC1=CC=CC2=C1C=CC=N2)CN1CCN(CC1)C(=O)C(C1=CC=CC=C1)C1=CC=CC=C1	experimental; investigational	Dofequidar
8.1581	[H][C@@]12CC[C@H](C(=O)COC(C)=O)[C@@]1(C)CC[C@@]1([H])[C@@]2([H])CCC2=CC(=O)CC[C@]12C	approved	Desoxycorticosterone acetate
8.1532	CC1=CC(N)=C2C=CC=CC2=[N+]1CCCCCCCCCC[N+]1=C(C)C=C(N)C2=C1C=CC=C2	approved; investigational	Dequalinium
8.1454	CC(C)(N)C(=O)N[C@H](CC1=CNC2=CC=CC=C12)C(=O)N[C@H](CC1=CNC2=CC=CC=C12)NC=O	approved; investigational	Macimorelin
8.1261	[H][C@@]12C[C@H]3OC(C)(C)O[C@@]3(C(=O)CCl)[C@@]1(C)C[C@H](O)[C@@]1(F)[C@@]2([H])CCC2=CC(=O)CC[C@]12C	approved; investigational; withdrawn	Halcinonide

8.1251	CC1=C(N(CC2=CC=C(OCCN3CCCCCC3)C=C2)C2=C1C=C(O)C=C2)C1=CC=C(O)C=C1	approved; investigational	Bazedoxifene
8.1077	[H][C@@]12CC3=CNC4=CC=CC(=C34)[C@@]1([H])C[C@H](CN2CC=C)C(=O)N(CCCN(C)C)C(=O)NCC	approved	Cabergoline
8.104	CN1CCC(CN2C3=CC=CC=C3SC3=CC=CC=C23)C1	approved	Methdilazine
8.0997	CN1CCC(CC1)=C1C2=C(CCC3=CC=CC=C13)SC=C2	approved	Pizotifen
8.0986	[H][C@]12CN3C=C(C(=O)NCC4=CC=C(F)C=C4F)C(=O)C(O)=C3C(=O)N1[C@H](C)CCO2	approved	Dolutegravir
8.0851	O=C1NC2=C(C=CC=C2)N1CCCN1CCN(CC1)C(C1=CC=CC=C1)C1=CC=CC=C1	approved; investigational	Oxatomide
8.0731	CCN1C(=O)N(CC2=CN=C3NC(CN4CCOCC4)=CC3=C12)C1=C(F)C(OC)=CC(OC)=C1F	approved; investigational	Pemigatinib
8.072	[H][C@@]12CC[C@](OC(=O)CCCCC)(C(C)=O)[C@@]1(C)CC[C@]1([H])[C@@]3([H])CCC(=O)C=C3CC[C@@]21[H]	approved	Gestonorone caproate
8.0546	CN1C2=C(C3=CC=CC=C13)C(=O)C(CN1C=CN=C1C)CC2	approved	Ondansetron
8.0504	OC1(N2CCN=C2C2=CC=CC=C12)C1=CC=C(Cl)C=C1	approved; investigational	Mazindol
8.0419	[H][C@@]1(C)C[C@@]2([H])[C@]3([H])C[C@]([H])(F)C4=CC(=O)C=C[C@]4(C)[C@@]3([H])[C@@]([H])(O)C[C@]2(C)[C@@]1([H])C(=O)CO	approved; withdrawn	Fluocortolone
8.0371	[H][C@]12CCCC3=C1C(=CC=C3)C(=O)N(C2)[C@@H]1CN2CCC1CC2	approved; investigational	Palonosetron
8.0194	[H][C@@]12OC3=C(O)C=CC4=C3[C@@]11CCN(C)[C@]([H])(C4)[C@]1(O)CCC2=O	approved; investigational; vet_approved	Oxymorphone
8.0015	CCS(=O)(=O)N1CC(CC#N)(C1)N1C=C(C=N1)C1=C2C=CNC2=NC=N1	approved; investigational	Baricitinib
7.9803	COC1=C(C=C2C(OC3=CC=C(NC(=O)NC4CC4)C(Cl)=C3)=CC=NC2=C1)C(N)=O	approved; investigational	Lenvatinib
7.9733	CC1=C(CCN2CCC(CC2)C2=NOC3=C2C=CC(F)=C3)C(=O)N2CCCCC2=N1	approved; investigational	Risperidone
7.9713	[H][C@]12CC[C@]([H])(C[C@@]([H])(C1)OC(=O)C1=CNC3=CC=CC=C13)N2C	approved; investigational	Tropisetron
7.9687	CN1CCCC(CC2C3=CC=CC=C3SC3=CC=CC=C23)C1	approved	Metixene

7.9556	CN1[C@H]2C[C@@H](C[C@@H]1[C@H]1O[C@@H]21)OC(=O)[C@H](CO)C1=CC=CC=C1	approved; investigational	Scopolamine
7.9499	CCC1=C(CC)C=C2CC(CC2=C1)NC[C@H](O)C1=C2C=CC(=O)NC2=C(O)C=C1	approved	Indacaterol
7.9453	NC(=O)C1=C2NCC[C@@H](C3CCN(CC3)C(=O)C=C)N2N=C1C1=CC=C(OC2=CC=CC=C2)C=C1	approved; investigational	Zanubrutinib
7.945	C[C@]12CC[C@H]3[C@@H](CCC4=CCCC[C@H]34)[C@@H]1CC[C@@]2(O)C#C	approved; investigational	Lynestrenol
7.9446	[H][C@]12CCN(CCCC(=O)C3=CC=C(F)C=C3)C[C@@]1([H])C1=CC=CC3=C1N2CCN3C	approved; investigational	Lumateperone
7.9331	ClC1=CC=CC(=C1)N1CCN(CCCN2N=C3C=CC=CN3C2=O)CC1	approved; investigational	Trazodone
7.9319	[H][C@]12[C@@H](C)C(S[C@]3([H])CN[C@@]([H])(C3)C(=O)NC3=CC=CC(=C3)C(O)=O)=C(N1C(=O)[C@]2([H])[C@@H](C)O)C(O)=O	approved; investigational	Ertapenem
7.9301	C[C@H]1C2[C@@H](OC1=O)[C@H](O)[C@]13[C@@H]4OC(=O)[C@]21O[C@@H]1OC(=O)[C@H](O)[C@]31[C@@H]([C@H]4O)C(C)(C)C	nutraceutical	ginkgolide-M
7.924	[H][C@@]12CC[C@@](O)(C#C)[C@@]1(CC)C=CC1=C3CCC(=O)C=C3CC[C@@]21[H]	approved	Gestrinone
7.919	[H][C@@]12C[C@@]3([H])[C@]4([H])C[C@H](F)C5=CC(=O)C=C[C@]5(C)[C@@]4(F)[C@@H](O)C[C@]3(C)[C@@]1(OC(C)(C)O2)C(=O)CO	approved; investigational; vet_approved	Fluocinolone acetonide
7.9144	[H][C@@]12C[C@@H](C)[C@H](C(=O)CO)[C@@]1(C)C[C@H](O)[C@@]1(Cl)[C@@]2([H])C[C@H](F)C2=CC(=O)C=C[C@]12C	approved	Clocortolone
7.9102	[H][C@@]12CC[C@](OC(=O)OCC)(C(=O)OCCl)[C@@]1(C)C[C@H](O)[C@@]1([H])[C@@]2([H])CCC2=CC(=O)C=C[C@]12C	approved	Loteprednol etabonate
7.9061	[H][C@@]12OC3=C(O)C=CC4=C3[C@@]11CCN(CC3CC3)[C@]([H])(C4)[C@]1(O)CCC2=C	approved; investigational; withdrawn	Nalmefene
7.8986	O[C@H]1CC[C@@]2(O)[C@H]3CC4=CC=C(O)C5=C4[C@@]2(CCN3CC2CCC2)[C@H]1O5	approved	Nalbuphine
7.8956	C[C@H]1COC2=C3N1C=C(C(O)=O)C(=O)C3=CC(F)=C2N1CCN(C)CC1	approved; investigational	Levofloxacin
7.8949	COC1=C(C=C(C=C1)C1=CC2=C(C=C1)C=C(C=C2)C(O)=O)C12CC3CC(CC(C3)C1)C2	approved	Adapalene
7.8934	CC1(C)O[C@@H]2C[C@H]3[C@@H]4C[C@H](F)C5=CC(=O)CC[C@]5(C)[C@H]4[C@@H](O)C[C@]3(C)[C@@]2(O1)C(=O)CO	approved	Flurandrenolide

7.8903	CC1=CC=C(NC(=O)C2(CC2)C2=CC=C3OC(F)(F)OC3=C2)N=C1C1=CC(=CC=C1)C(O)=O	approved	Lumacaftor
7.8878	[H][C@@]12CC[C@](O)(C(=O)CO)[C@@]1(C)C[C@H](O)[C@@]1([H])[C@@]2([H])C[C@H](C)C2=CC(=O)C=C[C@]12C	approved; vet_approved	Methylprednisolone
7.8867	NC(=O)C1=CC2=CC(=CC=C2O1)N1CCN(CCCCC2=CNC3=C2C=C(C=C3)C#N)CC1	approved	Vilazodone
7.8807	NC(=O)C([C@@H]1CCN(CCC2=CC3=C(OCC3)C=C2)C1)(C1=CC=CC=C1)C1=CC=CC=C1	approved; investigational	Darifenacin
7.8805	O[C@H](\C=C\[C@@H](C)[C@@]1([H])CC[C@@]2([H])\C(CCC[C@]12C)=C\C=C1\C[C@@H](O)C[C@H](O)C1=C)C1CC1	approved	Calcipotriol
7.868	[H][C@@]12[C@@H](C)C3=CC=CC(O)=C3C(=O)C1=C(O)[C@]1(O)C(=O)C(C(N)=O)=C(O)[C@@H](N(C)C)[C@]1([H])[C@H]2O	approved; investigational; vet_approved	Doxycycline
7.8669	[H][C@@]12C[C@@]3([H])C(=C(O)[C@]1(O)C(=O)C(C(N)=O)=C(O)[C@H]2N(C)C)C(=O)C1=C(O)C=CC(Cl)=C1[C@@]3(C)O	approved; investigational; vet_approved	Chlortetracycline
7.866	[H][C@@]12CCC[C@]1([H])C1=NN=C(CCN3CCN(CC3)C3=CC=CC(=C3)C(F)(F)F)N1C2	approved	Lorpiprazole
7.8606	CNC(=O)C1=C(SC2=CC=C3C(NN=C3\C=C\C3=CC=CC=N3)=C2)C=CC=C1	approved; investigational	Axitinib
7.8539	N#CC[C@H](C1CCCC1)N1C=C(C=N1)C1=C2C=CNC2=NC=N1	approved	Ruxolitinib
7.8442	CO\N=C(/C(=O)N[C@@H]1C(=O)N2[C@]1([H])SCC(C[N+]1(C)CCCC1)=C2C([O-])=O)C1=CSC(N)=N1	approved; investigational	Cefepime
7.8401	COC1=C(NC2=C3C=CC=CC3=NC3=CC=CC=C23)C=CC(NS(C)(=O)=O)=C1	approved; investigational	Amsacrine
7.8373	CN1CCC(CC1)=C1C2=NC=C(C=O)N2CCC2=CC=CC=C12	approved	Alcaftadine
7.827	CN1C2=C(C3=CC=CC=C13)C(=O)N(CC1=C(C)NC=N1)CC2	approved; withdrawn	Alosetron
7.8149	[H][C@@]12CC[C@](OC(=O)CCCCC)(C(C)=O)[C@@]1(C)CC[C@@]1([H])[C@@]2([H])CCC2=CC(=O)CC[C@]12C	approved; investigational	Hydroxyprogesterone caproate
7.8127	C[C@@H](C1=NC(=CS1)C1=CC=C(C=C1)C#N)[C@](O)(CN1C=NC=N1)C1=C(F)C=CC(F)=C1	approved; investigational	Isavuconazole
7.8044	[H][C@@]12CC[C@H](C(C)=O)[C@@]1(C)C[C@H](O)[C@@]1([H])[C@@]2([H])C[C@H](C)C2=CC(=O)CC[C@]12C	approved	Medrysone

7.7962	COC1=C(OC)C=C2C(N)=NC(=NC2=C1)N1CCN(CC1)C(=O)C1=CC=CO1	approved	Prazosin
7.7721	[H][C@@]12CC[C@](O)(C(=O)CO)[C@@]1(C)C[C@H](O)[C@@]1([H])[C@@]2([H])CCC2=CC(=O)CC[C@]12C	approved; vet_approved	Hydrocortisone
7.7704	CN1CCN(CC2=CC=C(C=C2)C(=O)NC2=CC(NC3=NC=CC(=N3)C3=CN=CC=C3)=C(C)C=C2)CC1	approved	Imatinib
7.762	CC1=NN(C(=O)\C1=N/NC1=CC=CC(C2=CC=CC(=C2)C(O)=O)=C1O)C1=CC=C(C)C(C)=C1	approved	Eltrombopag
7.7596	C(C=CC1=CC=CC=C1)N1CCN(CC1)C(C1=CC=CC=C1)C1=CC=CC=C1	approved; investigational	Cinnarizine
7.7551	C[C@H](NC1=C2N=CNC2=NC=N1)C1=CC2=CC=CC(Cl)=C2C(=O)N1C1=CC=CC=C1	approved; investigational	Duvelisib
7.7496	[H][C@@]12C[C@H](C)[C@](O)(C(=O)CCl)[C@@]1(C)C[C@H](O)[C@@]1(F)[C@@]2([H])C[C@H](F)C2=CC(=O)C=C[C@]12C	approved	Ulobetasol
7.7443	CC[C@H]1[C@@H]2C[C@H]3[C@@H]4N(C)C5=CC=CC=C5[C@]44C[C@@H](C2[C@H]4O)N3[C@@H]1O	approved; experimental	Ajmaline
7.7443	CC[C@H]1[C@@H]2C[C@H]3[C@@H]4N(C)C5=CC=CC=C5[C@]44C[C@@H](C2[C@H]4O)N3[C@@H]1O	approved; experimental	Ajmaline
7.7432	FC1=CC=C(CC2=NNC(=O)C3=CC=CC=C23)C=C1C(=O)N1CCN(CC1)C(=O)C1CC1	approved	Olaparib
7.7432	C[C@]12CC[C@H]3[C@@H](CCC4=CC(=O)CC[C@H]34)[C@@H]1CC[C@@H]2OS(O)(=O)=O	experimental; investigational	Nandrolone hydrogen sulfate
7.7429	COC1=CC=C2SCCN(CC2=C1)C(=O)CCN1CCC(CC2=CC=CC=C2)CC1	experimental; investigational	K201 free base
7.7404	CC1=CC(C)=C(C=C1)C(=O)OCC1=CC=C(C=C1)[C@@H](CN)C(=O)NC1=CC=C2C=NC=CC2=C1	approved	Netarsudil
7.7329	[H][C@@]12CC[C@](O)(C(=O)CO)[C@@]1(C)C[C@H](O)[C@@]1(F)[C@@]2([H])CCC2=CC(=O)CC[C@]12C	approved; investigational	Fludrocortisone
7.7253	OC1=C2N(C[C@H]3O[C@@H]4CC[C@@H](C4)N3C2=O)C=C(C(=O)NCC2=C(F)C=C(F)C=C2F)C1=O	approved; investigational	Bictegravir
7.7185	[H][C@@]1(OC2=C(O[C@@H]1CO)C=CC(=C2)[C@@]1([H])OC2=C(C(O)=CC(O)=C2)C(=O)[C@@H]1O)C1=CC(OC)=C(O)C=C1	experimental; investigational	Silibinin
7.7181	[H][C@@]12CCC(=O)[C@@]1(C)CC[C@@]1([H])[C@@]2([H])CCC2=C(O)C(=O)CC[C@]12C	approved; investigational; withdrawn	Formestane

7.7163	[H][C@]1(CNC(=O)C2=CC=C(Cl)S2)CN(C(=O)O1)C1=CC=C(C=C1)N1CCOCC1=O	approved	Rivaroxaban
7.7033	C[C@@H]1C(=O)O[C@H]2C[C@@]34[C@H]5C[C@@H](C(C)(C)C)[C@@]33[C@@H](O)C(=O)O[C@H]3O[C@@]4(C(=O)O5)[C@@]12O	nutraceutical	Ginkgolide A
7.6983	[H][C@@]12CC[C@@]3(CCC(=O)O3)[C@@]1(C)CC[C@@]1([H])[C@@]2([H])[C@@]([H])(CC2=CC(=O)CC[C@]12C)SC(C)=O	approved	Spironolactone
7.6921	NC1=NCC2N1C1=CC=CC=C1CC1=CC=CC=C21	approved; investigational	Epinastine
7.6699	[H][C@@]12C[C@@H](C)[C@H](C(=O)CO)[C@@]1(C)C[C@H](O)[C@@]1(F)[C@@]2([H])CCC2=CC(=O)C=C[C@]12C	approved	Desoximetasone
7.6677	[H][C@]12SC(C)(C)[C@@H](N1C(=O)[C@H]2NC(=O)C1=C(C)ON=C1C1=C(Cl)C=CC=C1F)C(O)=O	approved; investigational	Flucloxacillin
7.6596	O[C@H]1CCN(C1)C(=O)NC1=C2N=C(C=CN2N=C1)N1CCC[C@@H]1C1=C(F)C=CC(F)=C1	approved; investigational	Larotrectinib
7.6452	[H][C@@]12CN(C[C@]1([H])[C@H]2N)C1=NC2=C(C=C1F)C(=O)C(=CN2C1=C(F)C=C(F)C=C1)C(O)=O	approved; investigational; withdrawn	Trovafloxacin
7.6439	OC12CC3CC(C1)CC(C3)(C2)NCC(=O)N1CCC[C@H]1C#N	approved; investigational	Vildagliptin
7.6373	CCOCCN1C(=NC2=CC=CC=C12)C1CCN(CCC2=CC=C(C=C2)C(C)(C)C(O)=O)CC1	approved; investigational	Bilastine
7.6211	[H][C@@]12CC[C@](OC(=O)CC)(C(=O)COC(C)=O)[C@@]1(C)C[C@H](O)[C@@]1([H])[C@@]2([H])C[C@H](C)C2=CC(=O)C=C[C@]12C	approved; vet_approved	Methylprednisolone aceponate
7.6162	[H][C@]12SC(C)(C)[C@@H](N1C(=O)[C@H]2NC(=O)C(C(=O)OC1=CC2=C(CCC2)C=C1)C1=CC=CC=C1)C(O)=O	approved; investigational	Carindacillin
7.6028	ClC1=CC=CC=C1C(N1C=CN=C1)(C1=CC=CC=C1)C1=CC=CC=C1	approved; vet_approved	Clotrimazole
7.6022	CNC1=NC=C(C=C1C)C1=CC=C2C(=O)C(=CN(C3CC3)C2=C1C)C(O)=O	approved; investigational	Ozenoxacin
7.5739	OC1=CC=C2C(OC3=CC(O)=CC=C3C22OC(=O)C3=C2C=CC=C3)=C1	approved	Fluorescein
7.5713	[H][C@@]12C[C@@H](C)[C@](C)(C(=O)CC)[C@@]1(C)C[C@H](O)[C@@]1([H])[C@@]2([H])CCC2=CC(=O)C=C[C@]12C	approved	Rimexolone
7.5427	C1=CN(C=N1)C(C1=CC=CC=C1)C1=CC=C(C=C1)C1=CC=CC=C1	approved; investigational	Bifonazole

7.5349	[H][C@@]12CC[C@@](O)(C#C)[C@@]1(C)CC[C@]1([H])C3=C(CC[C@@]21[H])C=C(OC1CCCC1)C=C3	approved	Quinestrol
7.5347	[H][C@]12CC3=C(C(O)=C(O)C=C3)C3=CC=CC(CCN1C)=C23	approved; investigational	Apomorphine
7.5278	[H][C@@]1(CC[C@@]2([H])[C@]3([H])CC=C4C[C@@H](O)CC[C@]4(C)[C@@]3([H])CC[C@]12C)[C@H](C)CCCC(C)C	approved; investigational	Cholesterol
7.519	[H][C@@]12CC[C@@](O)(C#C)[C@@]1(CC)CC(=C)[C@]1([H])[C@@]3([H])CCCC=C3CC[C@@]21[H]	approved	Desogestrel
7.5103	[H][C@@]12C[C@@]3([H])[C@]4([H])C[C@H](F)C5=CC(=O)C=C[C@]5(C)[C@@]4([H])[C@@H](O)C[C@]3(C)[C@@]1(OC(C)(C)O2)C(=O)CO	approved; investigational	Flunisolide
7.5023	[H][C@]12CC3=C(NC4=CC=CC=C34)[C@H](N1C(=O)CN(C)C2=O)C1=CC2=C(OCO2)C=C1	approved; investigational	Tadalafil
7.4997	[H][C@@]12C[C@H](C)[C@](O)(C(=O)CO)[C@@]1(C)C[C@H](O)[C@@]1(F)[C@@]2([H])CCC2=CC(=O)C=C[C@]12C	approved; vet_approved	Betamethasone
7.4994	CN1CCCC(CC1)N1N=C(CC2=CC=C(Cl)C=C2)C2=CC=CC=C2C1=O	approved	Azelastine
7.4776	C[C@@H]1C(=O)O[C@H]2[C@H](O)[C@@]34[C@H]5C[C@@H](C(C)(C)C)[C@@]33[C@@H](O)C(=O)O[C@H]3O[C@@]4(C(=O)O5)[C@@]12O	nutraceutical	ginkgolide-B
7.4763	[H][C@]12SC(C)(C)[C@@H](N1C(=O)[C@H]2NC(=O)C1=C(C)ON=C1C1=CC=CC=C1)C(O)=O	approved; investigational	Oxacillin
7.4695	CN1N=NC(=N1)C1=NC=C(C=C1)C1=C(F)C=C(C=C1)N1C[C@H](CO)OC1=O	approved; investigational	Tedizolid
7.4662	[H][C@@]12CC[C@H](O)[C@@]1(C)CC[C@]1([H])C3=CC=C(OC(C)=O)C=C3CC[C@@]21[H]	approved; investigational; vet_approved	Estradiol acetate
7.4581	C[C@]12CC[C@H]3[C@@H](CCC4=CC(=O)CC[C@]34C)[C@@H]1CC[C@@H]2O	approved; investigational	Testosterone
7.4382	CCCC1=NC(=C(N1CC1=CC=C(C=C1)C1=C(C=CC=C1)C1=NN=NN1)C(O)=O)C(C)(C)O	approved; investigational	Olmesartan
7.4289	[H][C@@]12OC3=C(O)C=CC4=C3[C@@]11CCN(C)[C@]([H])(C4)[C@]1([H])C=C[C@@H]2O	approved; investigational	Morphine
7.4273	COC1=C(NC2=NC=CC(=N2)C2=CN(C)C3=C2C=CC=C3)C=C(NC(=O)C=C)C(=C1)N(C)CCN(C)C	approved	Osimertinib
7.4159	OC1=NC2=CC=CC=C2N1C1CCN(CCCC(=O)C2=CC=C(F)C=C2)CC1	approved; investigational	Benperidol
7.3939	[H][C@]12C[C@@H](O)C=C[C@]11CCN(C)CC3=C1C(O2)=C(OC)C=C3	approved	Galantamine

7.3903	[H][C@]1(C[C@@H]2CC[N@]1C[C@@H]2C=C)[C@H](O)C1=CC=NC2=CC=C(OC)C=C12	approved	Quinine
7.3866	OC1=C(CC2=C(O)C3=C(OC2=O)C=CC=C3)C(=O)OC2=C1C=CC=C2	approved	Dicoumarol
7.381	[H][C@@]12CC[C@@H](C)[C@]3([H])CC[C@@]4(C)OO[C@@]13[C@]([H])(O[C@H](OC)[C@@H]2C)O4	approved	Artemether
7.3783	ClC1=CC2=C(OC3=CC=CC=C3N=C2N2CCNCC2)C=C1	approved	Amoxapine
7.3704	[H][C@@]12CC[C@H](C(C)=O)[C@@]1(C)CC[C@@]1([H])[C@@]2([H])CCC2=CC(=O)CC[C@]12C	approved; vet_approved	Progesterone
7.3633	[H][C@@]1(CC[C@@]2([H])[C@]3([H])CCC4=CC(=O)CC[C@]4(C)[C@@]3([H])[C@@H](O)C[C@]12C=O)C(=O)CO	experimental; investigational	Aldosterone
7.3557	CCCS(=O)(=O)NC1=C(F)C(C(=O)C2=CNC3=NC=C(C=C23)C2=CC=C(Cl)C=C2)=C(F)C=C1	approved	Vemurafenib
7.3512	O=C(O[C@H]1CN2CCC1CC2)N1CCC2=CC=CC=C2[C@@H]1C1=CC=CC=C1	approved	Solifenacin
7.3425	[H][C@@]12C[C@H]3OC(C)(C)O[C@@]3(C(=O)CO)[C@@]1(C)C[C@H](O)[C@@]1([H])[C@@]2([H])CCC2=CC(=O)C=C[C@]12C	approved; investigational	Desonide
7.3287	OC1=CC=C(C=C1)C1(OC2=CC=CC=C2NC1=O)C1=CC=C(O)C=C1	approved	Bisoxatin
7.3164	ClC1=CC=CC(N2CCN(CCCCOC3=CC4=C(CCC(=O)N4)C=C3)CC2)=C1Cl	approved; investigational	Aripiprazole
7.316	[H][C@@]12CC[C@](O)(C(=O)OCCl)[C@@]1(C)C[C@H](O)[C@@]1([H])[C@@]2([H])CCC2=CC(=O)C=C[C@]12C	approved; experimental	Loteprednol
7.316	[H][C@@]12CC[C@](O)(C(=O)OCCl)[C@@]1(C)C[C@H](O)[C@@]1([H])[C@@]2([H])CCC2=CC(=O)C=C[C@]12C	approved; experimental	Loteprednol
7.3017	[H][C@@]12CC3=CN(C)C4=CC=CC(=C34)[C@@]1([H])C[C@@H](CNC(=O)OCC1=CC=CC=C1)CN2C	approved	Metergoline
7.2979	CN1CC[C@H](C[C@@H]1C1=NC2=CC=CC=C2N1)NC(=O)NC1=CC=C(C=C1)C#N	approved; investigational	Glasdegib
7.2891	COC1=CC=C(C=C1)N1N=C(C(N)=O)C2=C1C(=O)N(CC2)C1=CC=C(C=C1)N1CCCCC1=O	approved	Apixaban
7.279	[H][C@@]12CC[C@@H](C)[C@]3([H])CC[C@@]4(C)OO[C@@]13[C@]([H])(O[C@H](OCC)[C@@H]2C)O4	approved	Artemotil
7.2583	ClC1=C(CCN2CCN(CC2)C2=NSC3=CC=CC=C23)C=C2CC(=O)NC2=C1	approved	Ziprasidone

7.2484	[H][C@@]12CC3=C(C(O)=CC=C3N(C)C)C(=O)C1=C(O)[C@]1(O)C(=O)C(C(N)=O)=C(O)[C@@H](N(C)C)[C@]1([H])C2	approved; investigational	Minocycline
7.2426	[H][C@@]12OC3=C(OC)C=CC4=C3[C@@]11CCN(C)[C@]([H])(C4)[C@]1([H])CC=C2OC(=O)C1=CC=CC=C1	approved	Benzhydrocodone
7.2249	OC(COC1=CC=CC2=C1C(=O)C=C(O2)C(O)=O)COC1=CC=CC2=C1C(=O)C=C(O2)C(O)=O	approved	Cromoglicic acid
7.2085	[H][C@]12SC(C)(C)[C@@H](N1C(=O)[C@H]2NC(=O)C1=C(C)ON=C1C1=CC=CC=C1Cl)C(O)=O	approved; investigational; vet_approved	Cloxacillin
7.2022	OC1=CC=C2C[C@H]3N(CC=C)CC[C@@]45[C@@H](OC1=C24)C(=O)CC[C@@]35O	approved; vet_approved	Naloxone
7.201	CN1CCN(CCCN2C3=CC=CC=C3SC3=C2C=C(C=C3)C(F)(F)F)CC1	approved; investigational	Trifluoperazine
7.1968	[H][C@@]12CC[C@](O)(C(=O)CS)[C@@]1(C)C[C@H](O)[C@@]1([H])[C@@]2([H])CCC2=CC(=O)CC[C@]12C	approved; withdrawn	Tixocortol
7.1744	CC1=CC(CN2CCN(CC2)C(C2=CC=CC=C2)C2=CC=C(Cl)C=C2)=CC=C1	approved	Meclizine
7.1681	O=C(NC1CCN(CCC2=CNC3=C2C=CC=C3)CC1)C1=CC=CC=C1	approved; withdrawn	Indoramin
7.1594	COC1=C(Cl)C=C(CNC2=C(C=NC(=N2)N2CCC[C@H]2CO)C(=O)NCC2=NC=CC=N2)C=C1	approved	Avanafil
7.1499	[H][C@@]12CC3=CNC4=CC=CC(=C34)[C@@]1([H])C[C@@H](CSC)CN2CCC	approved; investigational; vet_approved; withdrawn	Pergolide
7.1329	CN1CCN(CC(=O)N2C3=CC=CC=C3C(=O)NC3=C2N=CC=C3)CC1	approved	Pirenzepine
7.124	CC1=CC(CN2CCC(CC2)=C2C3=CC=C(Cl)C=C3CCC3=C2N=CC=C3)=CN=C1	approved	Rupatadine
7.1227	CN1CCC(CC1)=C1C2=CC=CC=C2SC2=CC=CC=C12	approved	Pimethixene
7.0925	CN1C2=C(C=C(Cl)C=C2)C2(OC(C)=CC(=O)N2CC1=O)C1=CC=CC=C1	approved	Ketazolam
7.0904	O[C@H](C[C@H](O)\C=C\C1=C(C2=CC=C(F)C=C2)C2=CC=CC=C2N=C1C1CC1)CC(O)=O	approved	Pitavastatin
7.0842	[H][C@@]1(CC[C@@]2([H])[C@]3([H])CCC4=CC(O)=CC=C4[C@@]3([H])CC[C@]12C)OC(=O)CCC1CCCC1	approved; investigational; vet_approved	Estradiol cypionate

7.0783	CCCC(=O)C1=CC2=C(SC3=CC=CC=C3N2CCCN2CCN(C)CC2)C=C1	approved	Butaperazine
7.0737	O=C1C(CCS(=O)C2=CC=CC=C2)C(=O)N(N1C1=CC=CC=C1)C1=CC=CC=C1	approved	Sulfinpyrazone
7.0667	CCC12CCC3C(CCC4=CC(=O)CCC34)C1CCC2(O)C#C	approved	Norgestrel
7.0637	[H][C@@]12CCN(C[C@@H]1C=C)[C@]([H])(C2)[C@@H](O)C1=C2C=C(OC)C=CC2=NC=C1	approved; investigational	Quinidine
7.0545	CCOC1=CC=C(CC2=CC(=CC=C2Cl)[C@]23OC[C@](CO)(O2)[C@@H](O)[C@H](O)[C@H]3O)C=C1	approved; investigational	Ertugliflozin
7.0415	[H][C@@]12CC[C@@](O)(C#C)[C@@]1(C)CC[C@]1([H])[C@@]3([H])CCC(=O)C=C3CC[C@@]21[H]	approved	Norethisterone
7.0403	[18F]C1=CC=C(C=N1)C1=CC=C2C(NC3=C2C=NC=C3)=C1	approved; investigational	Flortaucipir F-18
7.0324	[H][C@]12SC(C)(C)[C@@H](N1C(=O)[C@H]2N1C(=O)[C@H](NC1(C)C)C1=CC=CC=C1)C(O)=O	approved; vet_approved; withdrawn	Hetacillin
7.0269	CN1CCCCC1CCN1C2=C(SC3=C1C=C(C=C3)S(C)=O)C=CC=C2	approved; investigational	Mesoridazine
7.0153	CC1=NN=C2CN=C(C3=CC=CC=C3Cl)C3=C(C=CC(Cl)=C3)N12	approved; investigational	Triazolam
7.0054	[H][C@@]12C[C@@H](C)[C@H](C(=O)CO)[C@@]1(C)C[C@H](O)[C@@]1(F)[C@@]2([H])C[C@H](F)C2=CC(=O)C=C[C@]12C	approved; investigational; withdrawn	Difluocortolone
6.9953	OC(C1=CC=CC=C1)(C1=CC=CC=C1)C12CC[N+](CCOCC3=CC=CC=C3)(CC1)CC2	approved	Umeclidinium
6.9911	CN(C)C1=CC=C(C=C1)[C@H]1C[C@@]2(C)[C@@H](CC[C@]2(O)C(C)=O)[C@@H]2CCC3=CC(=O)CCC3=C12	approved	Ulipristal
6.9817	OC(CCN1CCCCC1)(C1CC2CC1C=C2)C1=CC=CC=C1	approved; investigational	Biperiden
6.9798	[H][C@@]12C[C@@H](O)[C@](O)(C(=O)CO)[C@@]1(C)C[C@H](O)[C@@]1(F)[C@@]2([H])CCC2=CC(=O)C=C[C@]12C	approved; vet_approved	Triamcinolone
6.9737	CN(C)C(=O)C(CCN1CCC(O)(CC1)C1=CC=C(Cl)C=C1)(C1=CC=CC=C1)C1=CC=CC=C1	approved	Loperamide
6.9407	C#CC1=CC=CC(NC2=NC=NC3=CC4=C(OCCOCCOCCO4)C=C23)=C1	experimental; investigational	Icotinib
6.9219	CN1CCC2=C(C1)C(C1=CC=CC=C21)C1=CC=CC=C1	approved	Phenindamine

6.9192	OC1=CC=C(C=C1)C1=C(C(=O)C2=CC=C(OCCN3CCCCC3)C=C2)C2=C(S1)C=C(O)C=C2	approved; investigational	Raloxifene
6.8993	[H][C@]12SC(C)(C)[C@@H](N1C(=O)[C@H]2NC(=O)C1=C(C)ON=C1C1=C(Cl)C=CC=C1Cl)C(O)=O	approved; investigational; vet_approved	Dicloxacillin
6.8929	[H][C@]1(O)CC[C@@]2([H])[C@]3([H])CCC4=CC(OC(=O)C5=CC=CC=C5)=CC=C4[C@@]3([H])CC[C@]12C	approved; investigational; vet_approved	Estradiol benzoate
6.8903	C[C@@H]1C(=O)O[C@H]2[C@H](O)[C@]34[C@@H]5OC(=O)[C@]3(O[C@@H]3OC(=O)[C@H](O)[C@]43[C@@H]([C@H]5O)C(C)(C)C)[C@@]12O	nutraceutical	ginkgolide-C
6.8733	[H][C@@]12CCC(=O)[C@@]1(C)CC[C@]1([H])C3=C(CC[C@@]21[H])C=C(O)C=C3	approved	Estrone
6.8313	COC1=C(OC)C=C2C(N)=NC(=NC2=C1)N1CCN(CC1)C(=O)C1CCCO1	approved	Terazosin
6.8155	CC(=O)OC1=CC2=C(CCN(C2)C(C(=O)C2CC2)C2=CC=CC=C2F)S1	approved	Prasugrel
6.802	OCCN1CCN(CCCN2C3=CC=CC=C3SC3=C2C=C(Cl)C=C3)CC1	approved	Perphenazine
6.7622	CC1=NC=C(OC[C@]2(C[C@H]2C(=O)NC2=NC=C(F)C=C2)C2=CC=CC(F)=C2)C(C)=N1	approved; investigational	Lemborexant
6.7604	[H][C@]12SC(C)(C)[C@@H](N1C(=O)[C@H]2NC(=O)C1=C(OCC)C=CC2=CC=CC=C12)C(O)=O	approved; investigational	Nafcillin
6.7564	[H][C@]12C[C@@]3([H])[C@H](N(C)C)C(O)=C(C(N)=O)C(=O)[C@@]3(O)C(O)=C1C(=O)C1=C([C@H]2O)C(Cl)=CC=C1O	approved	Demeclocycline
6.7518	FC1=CC=C(C=C1)C(N1CCN(C\C=C\C2=CC=CC=C2)CC1)C1=CC=C(F)C=C1	approved	Flunarizine
6.7423	COC(=O)N(C)C1=C(N)N=C(N=C1N)C1=NN(CC2=C(F)C=CC=C2)C2=C1C=CC=N2	approved	Riociguat
6.7315	CN1CCC(CC1)=C1C2=CC=CC=C2C=CC2=CC=CC=C12	approved	Cyproheptadine
6.6953	FC1=CC=C(C=C1)[C@@H]1CCNC[C@H]1COC1=CC2=C(OCO2)C=C1	approved; investigational	Paroxetine
6.6944	CN(C1CCN(CC1)C1=NC2=C(C=CC=C2)N1CC1=CC=C(F)C=C1)C1=NC=CC(=O)N1	approved; investigational	Mizolastine
6.6555	[H][C@]1(CC[C@H](O)C2=CC=C(F)C=C2)C(=O)N(C2=CC=C(F)C=C2)[C@]1([H])C1=CC=C(O)C=C1	approved	Ezetimibe
6.6444	CC(C)NC1=C(N=CC=C1)N1CCN(CC1)C(=O)C1=CC2=C(N1)C=CC(NS(C)(=O)=O)=C2	approved	Delavirdine

6.6379	CN1CCC(CC1)=C1C2=CC=CC=C2CCC2=C1N=CC=C2	approved	Azatadine
6.6355	CN1CCN(CC1)C1=NC2=CC=CC=C2OC2=C1C=C(Cl)C=C2	approved	Loxapine
6.6278	CN1CCN(CC1)C1=NC2=CC=CC=C2NC2=C1C=C(C)S2	approved; investigational	Olanzapine
6.5803	NS(=O)(=O)C1=C(Cl)C=C2NC(NS(=O)(=O)C2=C1)C1CC2CC1C=C2	approved	Cyclothiazide
6.5563	CC(C)(C1=CC=C(OCC2CO2)C=C1)C1=CC=C(OCC2CO2)C=C1	approved; experimental	Bisphenol A diglycidyl ether
6.5563	CC(C)(C1=CC=C(OCC2CO2)C=C1)C1=CC=C(OCC2CO2)C=C1	approved; experimental	Bisphenol A diglycidyl ether
6.5176	OC(=O)C1=CC=C(C=C1)N1N=C(N=C1C1=CC=CC=C1O)C1=CC=CC=C1O	approved; investigational	Deferasirox
6.5075	CN1CC[C@@H]([C@H](O)C1)C1=C(O)C=C(O)C2=C1OC(=CC2=O)C1=CC=CC=C1Cl	experimental; investigational	Alvocidib
6.4748	C1CN2CCC1C(C2)N1C2=CC=CC=C2CCC2=CC=CC=C12	approved	Quinupramine
6.4663	CNCC1=CC=C(C=C1)C1=C2CCNC(=O)C3=C2C(N1)=CC(F)=C3	approved; investigational	Rucaparib
6.4529	CN1CCN2C(C1)C1=CC=CC=C1CC1=CC=CC=C21	approved; investigational	Mianserin
6.4341	CC1=C(CCN2CCC(CC2)C2=NOC3=C2C=CC(F)=C3)C(=O)N2CCCC(O)C2=N1	approved	Paliperidone
6.3676	[H][C@@]12CCCC[C@@]11CCN(C)[C@@H]2CC2=C1C=C(O)C=C2	approved	Levorphanol
6.3649	[H][C@@]12CC[C@@](O)(CC#N)[C@@]1(C)CCC1=C3CCC(=O)C=C3CC[C@@]21[H]	approved	Dienogest
6.3632	CC1=C2NC(=O)C3=C(N=CC=C3)N(C3CC3)C2=NC=C1	approved	Nevirapine
6.3625	[H][C@@]12CC[C@](C)(C(C)=O)[C@@]1(C)CC[C@@]1([H])[C@@]2([H])C=C(C)C2=CC(=O)CC[C@]12C	approved; withdrawn	Medrogestone
6.3508	COC1=CC=C2C=C3C4=CC5=C(OCO5)C=C4CC[N+]3=CC2=C1OC	approved; investigational	Berberine
6.34	FC1=CC=C(C=C1)C(CCCN1CCC2(CC1)N(CNC2=O)C1=CC=CC=C1)C1=CC=C(F)C=C1	approved; investigational	Fluspirilene
6.3298	O=C(C1CCCCC1)N1CC2N(CCC3=CC=CC=C23)C(=O)C1	approved; investigational; vet_approved	Praziquantel

6.2997	[H][C@@]12C[C@@]3([H])C(=C(O)[C@]1(O)C(=O)C(C(N)=O)=C(O)[C@H]2N(C)C)C(=O)C1=C(O)C=CC=C1[C@@]3(C)O	approved; vet_approved	Tetracycline
6.272	[H][C@@]12CC[C@H](C(C)=O)[C@@]1(C)CC[C@]1([H])[C@@]2([H])C=CC2=CC(=O)CC[C@@]12C	approved; investigational; withdrawn	Dydrogesterone
6.2678	[H][C@@]12CCC(=O)[C@@]1(C)CC[C@@]1([H])[C@@]2([H])CC=C2C[C@@]([H])(O)CC[C@]12C	approved; investigational; nutraceutical	Prasterone
6.2678	[H][C@@]12CCC(=O)[C@@]1(C)CC[C@@]1([H])[C@@]2([H])CC=C2C[C@@]([H])(O)CC[C@]12C	approved; investigational; nutraceutical	Prasterone
6.2123	[H][C@@]12CC[C@H](O)[C@H](C(=O)OC)[C@@]1([H])C[C@]1([H])N(CCC3=C1NC1=CC=CC=C31)C2	approved; investigational; vet_approved	Yohimbine
6.2005	CCN1CC(CCN2CCOCC2)C(C1=O)(C1=CC=CC=C1)C1=CC=CC=C1	approved; vet_approved	Doxapram
6.1782	C[N+]1(C)[C@H]2C[C@@H](C[C@@H]1[C@H]1O[C@@H]21)OC(=O)[C@H](CO)C1=CC=CC=C1	approved	Methscopolamine
6.1565	[H][C@]12COC(=O)[C@]1([H])[C@H](C1=CC(OC)=C(OC)C(OC)=C1)C1=CC3=C(OCO3)C=C1[C@@H]2O	approved	Podofilox
6.1508	CN1CCN2C(C1)C1=CC=CC=C1CC1=C2N=CC=C1	approved	Mirtazapine
6.1103	CC(C)(C)C1=CC(=CC=C1N1CCCC1)C1=CC(=CC=C1OCCO)C1=CC=C(C=C1)C(O)=O	approved; investigational	Trifarotene
6.0901	OC1=CC=C(C=C1)C1(OC(=O)C2=CC=CC=C12)C1=CC=C(O)C=C1	approved; withdrawn	Phenolphthalein
5.9564	[H][C@@]12CCCC[C@@]11CCN(CC=C)[C@@H]2CC2=C1C=C(O)C=C2	approved	Levallorphan
5.8934	COC1=C(OC)C=C2[C@@H](CN(C)CCCN3CCC4=CC(OC)=C(OC)C=C4CC3=O)CC2=C1	approved	Ivabradine
5.7974	[H][C@]12CC[C@]([H])(C[C@@]([H])(C1)OC(C1=CC=CC=C1)C1=CC=CC=C1)N2C	approved	Benzatropine
5.7743	CC(C)N=C1C=C2N(C3=CC=C(Cl)C=C3)C3=C(C=CC=C3)N=C2C=C1NC1=CC=C(Cl)C=C1	approved; investigational	Clofazimine
5.7607	COC1=C(OC)C=C2C(=O)C(CC3CCN(CC4=CC=CC=C4)CC3)CC2=C1	approved	Donepezil
5.729	OC(CNCC(O)C1CCC2=C(O1)C=CC(F)=C2)C1CCC2=C(O1)C=CC(F)=C2	approved; investigational	Nebivolol
5.7121	[H][C@]12CCCC[C@]11CCN(C)[C@H]2CC2=C1C=C(OC)C=C2	approved	Dextromethorphan

5.5985	[H][C@@]12C[C@]1([H])[C@@]1([H])[C@]3([H])[C@]4([H])C[C@]4([H])[C@@]4(CCC(=O)O4)[C@@]3(C)CC[C@]1([H])[C@@]1(C)CCC(=O)C=C21	approved	Drospirenone
5.4482	C[N@@+]12CC[C@@H](CC1)C(C2)OC(=O)C(O)(C1=CC=CC=C1)C1=CC=CC=C1	approved	Clidinium
5.4266	CN1CCC[C@@H]1CC1=CNC2=C1C=C(CCS(=O)(=O)C1=CC=CC=C1)C=C2	approved; investigational	Eletriptan
5.2269	C1C2CNCC1C1=C2C=C2N=CC=NC2=C1	approved; investigational	Varenicline
4.941	ClC1=CC2=C(C=C1)C(=C1CCNCC1)C1=C(CC2)C=CC=N1	approved; investigational	Desloratadine
4.09	CC(=O)OC1=CC=C(C=C1)C1(C(=O)NC2=CC=CC=C12)C1=CC=C(OC(C)=O)C=C1	approved; investigational; withdrawn	Oxyphenisatin

Table S5. Isomeric SMILE and pIC50 for the molecules studied in this work.

Molecule	pKIC50
Acebutolol CCCC(=O)NC1=CC(=C(C=C1)OCC(CNC(C)C)O)C(=O)C	6.14
Alprenolol CC(C)NCC(COC1=CC=CC=C1CC=C)O	8.94
Aspirin CC(=O)OC1=CC=CC=C1C(=O)O	8.49
Betaxolol CC(C)NCC(COC1=CC=C(C=C1)CCOCC2CC2)O	7.43
Captopril C[C@H](CS)C(=O)N1CCC[C@H]1C(=O)O	9.92
Carvedilol COC1=CC=CC=C1OCCNCC(COC2=CC=CC3=C2C4=CC=CC=C4N3)O	9.75
Cilostazol C1CCC(CC1)N2C(=NN=N2)CCCCOC3=CC4=C(C=C3)NC(=O)CC4	8.41
Cinnarizine C1CN(CCN1C/C=C/C2=CC=CC=C2)C(C3=CC=CC=C3)C4=CC=CC=C4	6.79
Clevidipine CCCC(=O)OCOC(=O)C1=C(NC(=C(C1C2=C(C(=CC=C2)Cl)Cl)C(=O)OC)C)C	8.15
Clonidine C1CN=C(N1)NC2=C(C=CC=C2Cl)Cl	8.70
Diltiazem CC(=O)O[C@@H]1[C@@H](SC2=CC=CC=C2N(C1=O)CCN(C)C)C3=CC=C(C=C3)OC	7.55
Dipyridamole C1CCN(CC1)C2=NC(=NC3=C2N=C(N=C3N4CCCCC4)N(CCO)CCO)N(CCO)CCO	7.82
Doxazosin COC1=C(C=C2C(=C1)C(=NC(=N2)N3CCN(CC3)C(=O)C4COC5=CC=CC=C5O4)N)OC	9.13
Enalapril CCOC(=O)[C@H](CCC1=CC=CC=C1)N[C@@H](C)C(=O)N2CCC[C@H]2C(=O)O	8.92
Eprosartan CCCCC1=NC=C(N1CC2=CC=C(C=C2)C(=O)O)/C=C(\CC3=CC=CS3)/C(=O)O	8.70
Esmolol CC(C)NCC(COC1=CC=C(C=C1)CCC(=O)OC)O	6.71
Felodipine CCOC(=O)C1=C(NC(=C(C1C2=C(C(=CC=C2)Cl)Cl)C(=O)OC)C)C	7.64
Flunarizine C1CN(CCN1C/C=C/C2=CC=CC=C2)C(C3=CC=C(C=C3)F)C4=CC=C(C=C4)F	7.10

Fosinopril<br>CCC(=O)OC(C(C)C)OP(=O)(CCCCC1=CC=CC=C1)CC(=O)N2C[C@@H](C[C@H]2C(=O)O)C3CCCCC3	9.00
Gallopamil 1<br>CC(C)C(CCCN(C)CCC1=CC(=C(C=C1)OC)OC)(C#N)C2=CC(=C(C(=C2)OC)OC)OC	7.96
Hydralazine<br>C1=CC=C2C(=C1)C=NN=C2NN	6.05
Irbesartan<br>CCCCC1=NC2(CCCC2)C(=O)N1CC3=CC=C(C=C3)C4=CC=CC=C4C5=NNN=N5	8.05
Isradipine<br><br>CC1=C(C(C(=C(N1)C)C(=O)OC(C)C)C2=CC=CC3=NON=C32)C(=O)OC	8.66
Labetalol<br>CC(CCC1=CC=CC=C1)NCC(C2=CC(=C(C=C2)O)C(=O)N)O	8.00
Levamlodipine<br>CCOC(=O)C1=C(NC(=C([C@@H]1C2=CC=CC=C2Cl)C(=O)OC)C)COCCN	8.70
Lidoflazine<br>CC1=C(C(=CC=C1)C)NC(=O)CN2CCN(CC2)CCCC(C3=CC=C(C=C3)F)C4=CC=C(C=C4)F	7.80
Lisinopril<br>C1C[C@H](N(C1)C(=O)[C@H](CCCCN)N[C@@H](CCC2=CC=CC=C2)C(=O)O)C(=O)O	10.00
Metoprolol<br>CC(C)NCC(COC1=CC=C(C=C1)CCOC)O	6.75
Milrinone<br>CC1=C(C=C(C(=O)N1)C#N)C2=CC=NC=C2	6.91
Moexipril<br>CCOC(=O)[C@H](CCC1=CC=CC=C1)N[C@@H](C)C(=O)N2CC3=CC(=C(C=C3C[C@H]2C(=O)O)OC)OC	8.59
Nebivolol<br>C1CC2=C(C=CC(=C2)F)OC1C(CNCC(C3CCC4=C(O3)C=CC(=C4)F)O)O	6.50
Nifedipine<br>CC1=C(C(C(=C(N1)C)C(=O)OC)C2=CC=CC=C2[N+](=O)[O-])C(=O)OC	9.00
Nisoldipine<br>CC1=C(C(C(=C(N1)C)C(=O)OCC(C)C)C2=CC=CC=C2[N+](=O)[O-])C(=O)OC	10.82
Nitroglycerin<br>C(C(CO[N+](=O)[O-])O[N+](=O)[O-])O[N+](=O)[O-]	7.23
Olmesartan<br>CCCC1=NC(=C(N1CC2=CC=C(C=C2)C3=CC=CC=C3C4=NNN=N4)C(=O)O)C(C)(C)O	8.11
Papaverine<br>COC1=C(C=C(C=C1)CC2=NC=CC3=CC(=C(C=C32)OC)OC)OC	7.77
Perhexiline<br>C1CCC(CC1)C(CC2CCCCN2)C3CCCCC3	5.98
Perindopril<br>CCC[C@@H](C(=O)OCC)N[C@@H](C)C(=O)N1[C@H]2CCCC[C@H]2C[C@H]1C(=O)O	8.82
Phenoxybenzamine<br>CC(COC1=CC=CC=C1)N(CCCl)CC2=CC=CC=C2	5.57
Pindolol<br>CC(C)NCC(COC1=CC=CC2=C1C=CN2)O	9.26
Prazosin<br>COC1=C(C=C2C(=C1)C(=NC(=N2)N3CCN(CC3)C(=O)C4=CC=CO4)N)OC	9.77
Prenylamine<br>CC(CC1=CC=CC=C1)NCCC(C2=CC=CC=C2)C3=CC=CC=C3	7.19
Quinapril<br>CCOC(=O)[C@H](CCC1=CC=CC=C1)N[C@@H](C)C(=O)N2CC3=CC=CC=C3C[C@H]2C(=O)O	8.08
Ramipril<br>CCOC(=O)[C@H](CCC1=CC=CC=C1)N[C@@H](C)C(=O)N2[C@H]3CCC[C@H]3C[C@H]2C(=O)O	8.40
Sildenafil<br>CCCC1=NN(C2=C1N=C(NC2=O)C3=C(C=CC(=C3)S(=O)(=O)N4CCN(CC4)C)OCC)C	9.30
Sotalol<br>CC(C)NCC(C1=CC=C(C=C1)NS(=O)(=O)C)O	6.36
Telmisartan	9.48

CCCC1=NC2=C(N1CC3=CC=C(C=C3)C4=CC=CC=C4C(=O)O)C=C(C=C2C)C5=NC6=C C=CC=C6N5C	
Terazosin<br>COC1=C(C=C2C(=C1)C(=NC(=N2)N3CCN(CC3)C(=O)C4CCCO4)N)OC	7.77
Tiapamil<br>CN(CCCC1(S(=O)(=O)CCCS1(=O)=O)C2=CC(=C(C=C2)OC)OC)CCC3=CC(=C(C=C3)O C)OC	4.87
Trandolapril<br>CCOC(=O)[C@H](CCC1=CC=CC=C1)N[C@@H](C)C(=O)N2[C@H]3CCCC[C@@H]3C[C@H]2C(=O)O	9.03
Valsartan<br>CCCCC(=O)N(CC1=CC=C(C=C1)C2=CC=CC=C2C3=NNN=N3)[C@@H](C(C)C)C(=O)O	8.57
Vardenafil<br>CCCC1=NC(=C2N1N=C(NC2=O)C3=C(C=CC(=C3)S(=O)(=O)N4CCN(CC4)CC)OCC)C	10.54
Vinpocetine<br>CC[C@@]12CCCN3[C@@H]1C4=C(CC3)C5=CC=CC=C5N4C(=C2)C(=O)OCC	7.41
Zofenopril<br>C[C@H](CSC(=O)C1=CC=CC=C1)C(=O)N2C[C@H](C[C@H]2C(=O)O)SC3=CC=CC=C3	9.40
